# Research Status of Sarcosaprophagous Beetles as Forensic Indicators

**DOI:** 10.3390/insects15090711

**Published:** 2024-09-17

**Authors:** Shipeng Shao, Siqi Liu, Liangliang Li, Gengwang Hu, Yingna Zhang, Yu Wang

**Affiliations:** 1 Department of Forensic Medicine, Soochow University, Ganjiang East Road, Suzhou 215000, China; 20224221080@stu.suda.edu.cn (S.S.); 20234221084@stu.suda.edu.cn (S.L.); 20214221010@stu.suda.edu.cn (G.H.); 2 Key Laboratory of Evidence Identification in Universities of Shandong Province, Shandong University of Political Science and Law, Jiefang East Road, Jinan 250014, China; 002325@sdupsl.edu.cn; 3 Department of Anatomy, Shihezi University School of Medicine, Shihezi 832000, China

**Keywords:** Coleoptera, forensics, carcass, postmortem interval, sarcosaprophagous

## Abstract

**Simple Summary:**

As an important group of species in forensic entomology, sarcosaprophagous beetles can provide valuable clues to the postmortem interval of cadavers, especially in the late stages of decay. However, compared with flies, these species have not received much attention, and their potential value in forensic science is yet to be fully exploited. Therefore, in order to attract people’s attention to them and to increase their use in forensic practice, we conducted a bibliometric analysis of the literature on sarcosaprophagous beetles, statistically analyzed the beetles mentioned in successions, cases, and other experiments in relation to cadavers, briefly introduced each family of sarcosaprophagous beetles, and discussed the value of their immature species identification and forensic postmortem interval estimation.

**Abstract:**

In forensic entomology, research focused on sarcosaprophagous flies, but the sarcosaprophagous beetles, as important “forensic indicator species” of late-stage PMI in cadaver decomposition, received less attention. To increase attention on, and use and understanding of, sarcosaprophagous beetles in forensic entomology, this paper presents a bibliometric analysis of the available relevant literature. The occurrence frequency of beetle families and species from succession studies, actual cases, and experiments were calculated and illustrated using graphs. As a result, a total of 14 families and 1077 species associated with carcasses were collected, with Staphylinidae being the most frequently recorded among the families, and *Necrobia rufipes* (Fabricius, 1781) (Coleoptera: Cleridae) being the most frequently recorded species. In addition, a brief introduction of the cadaver-related beetles of each family is given, and research on the species identification of the immature stages, age estimation of the immature stages, and estimation of the arrival time of sarcosaprophagous beetles are discussed and prospected. This work will aid in the increased use of sarcosaprophagous beetles in forensic science practice in the future.

## 1. Introduction

Beetles are a general term for insects of the order Coleoptera. They are characterized by complete metamorphosis, where adults have a thick and sclerotized forewing and usually a pair of membranous hind wings. The Coleoptera includes four suborders, 166 families, and about 380,000 described species, making it the largest order in the animal kingdom, accounting for about 40% of the described insect diversity [1,2]. They are found in all parts of the world except the oceans, feeding mostly on animals and plants, but also on decomposing matter [3,4]. Beetles play a complex and two-sided role in natural ecosystems and human activities. For example, some beetle species destroy crops, wood, textiles, stored food, and transmit parasites and diseases [5], while others can also provide benefits to humans by contributing to crop pollination, nutrient cycling, soil aeration, and maintaining ecological balance [6,7]. In addition, some cadaver-associated beetles can play an important role in providing answers to questions in legal investigations, especially in estimating the postmortem interval (PMI) [8,9,10,11].

Forensic entomology is a science that uses the theories and techniques of entomology and other natural sciences to study insects and other arthropods related to certain criminal events or proceedings in order to provide clues and evidence for judicial practice [12]. Most often, forensically relevant insects are used to estimate the PMI, either by using known patterns of insect succession on cadavers or regularities in insect development. More specifically, using models of insect succession on cadavers under given environmental conditions and a set of insect taxa found at the scene of death, we can try to estimate the range of PMI. Alternatively using the close relationship between temperature and developmental rate allows the creation of insect developmental models for estimating the minimum PMI (PMI_min_) [13]. With the improvement of these developmental models in recent years, beetles are becoming more commonly used in criminal investigations.

At present, forensic research is mainly concentrated on flies, and many sarcosaprophagous flies are studied using morphology, taxonomy, development, and molecular aspects. Comparably, sarcosaprophagous beetles received less attention. For example, some forensic studies focused on the patterns of colonization of Diptera, while ignoring Coleopteran succession [14,15,16,17]. Dipterans colonize carcasses in the early stages of decomposition, where the Calliphoridae can appear on cadavers in the first hours or even minutes after death, with their larvae forming large aggregates during feeding [13,18,19]. Unlike the Diptera, the Coleoptera are active in the later stages of decay, or even in the skeletonization stage and can thus compensate for the absence of fly indicators to become important “forensic indicators” for estimating PMI_min_ in the late stage of cadaver decomposition [20]. There are exceptions, however, where some carrion beetles (Coleoptera: Staphylinidae), such as *Thanatophilus micans* (Fabricius, 1794) can reach the body within 24 h of death [21]. They, similar to flies, can be good indicators of PMI in the early to mid-stages of cadaver decay.

Sarcosaprophagous beetle research can consolidate and strengthen PMI estimates in early and middle cadaver decay stages, alongside fly-based estimates, while also further improving and expanding the time window for PMI estimation. When the cadaver enters the “post-fly phase”, or in some situations that are not suitable for flies colonization, such as winter [22,23], beetles become the most valuable PMI indicators on the cadaver. Unfortunately, research on sarcosaprophagous beetles is limited, with no data available for many species. In order to shed light on this undervalued group of insects, this paper reviewed the relevant forensic research and records and composed a brief introduction for each sarcosaprophagous beetle family, followed by a summary of the research. This includes the species and frequency of occurrence on cadavers and the research progress in terms of species identification during the immature stages, age estimation of the immature stages, and estimation of the time of arrival at the cadaver. This work will serve to increase the use of beetle evidence in forensic entomology.

## 2. Materials and Methods

**Figure 1:**
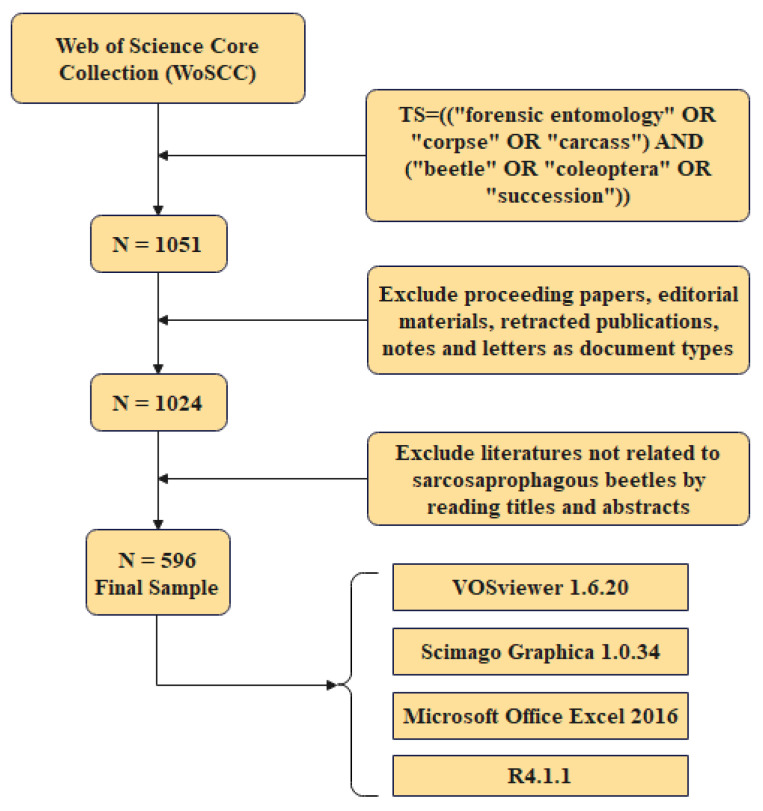
Flow chart illustrating literature collection, screening and analysis of cadaver-associated beetle research and reports.

The Science Citation Index Expanded (SCI-EXPANDED) and Social Sciences Citation Index (SSCI) databases of the Web of Science Core Collection (WoSCC) were utilized to search for insect succession studies, cases, and experiments involving cadaver-associated beetles. The search formula used was TS = ((“forensic entomology” OR “corpse” OR “carcass”) AND (“beetle” OR “Coleoptera” OR “succession”)). The search excluded proceedings papers, editorial materials, retracted publications, notes, and letters as document types. Literature that was not related to sarcosaprophagous beetles was excluded by reading titles and abstracts. Publication data were gathered on 20 March 2024 (Figure 1).

The search results were exported in plain text format, and the full record and cited references of the documents were collected. This included information such as country, keywords and other relevant details. Based on this data, VOSviewer [24] was used to construct the co-occurrence network of author keywords, and before that, the keywords were disambiguated (merging singular and plural forms and synonyms in keywords and deleting irrelevant keywords). Scimago Graphica [25] was used to build a world map to visualize the distribution and the number of publicatio ns and the collaboration between countries/regions. In order to estimate the number of beetle species associated with cadavers and to obtain more useful information, we also used Microsoft Excel 2016 (Microsoft, Washington, DC, USA) and R4.1.1 (R Core Team 2013; Vienna, Austria) to record the beetle species, carcass or bait type, beetle diet preference, and occurrence frequency based on the data collected from the final 596 pieces of literature (including various succession experiments, experiments using cadavers or traps to find or collect insects, and case reports).

## 3. Results and Discussion

### 3.1. Co-Occurrence Network of Author Keywords

**Figure 2:**
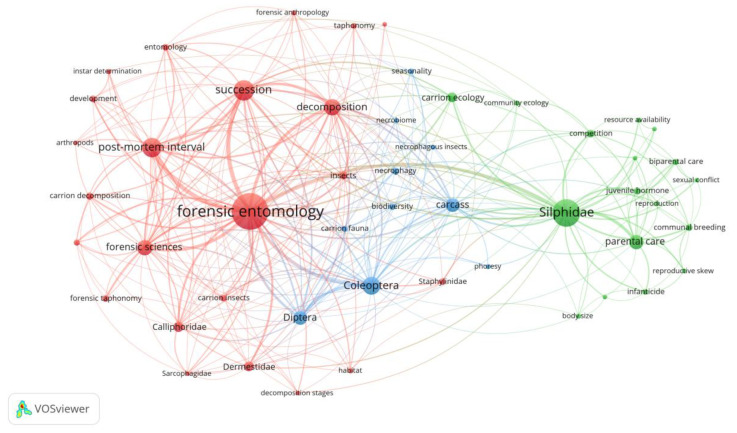
The network map of author keywords for studies on cadaver-associated beetles.

After disambiguation treatment, 50 keywords with frequencies greater than or equal to five were found (Supplementary Table S1). Then, visual analysis was performed on these 50 keywords (Figure 2). The cluster analysis resulted in three clusters, where those in red and blue mainly include insect succession studies, as well as studies on development and corpse ecology from a forensic perspective. The green cluster includes entomology, the study of insect physiology, behavioral ecology, and evolutionary biology. “Forensic entomology”, as the largest node, is at the heart of the network, closely linked to keywords such as “succession”, “post-mortem interval”, and “Silphidae”. This means that it is the most frequently mentioned topic in the literature on cadaver-associated beetles. Insect succession is probably one of the most focused topics in forensic entomology and is the focus of several research papers, perhaps because of its relevance to solving forensic problems, especially PMI estimation. The frequent occurrence of the keyword “post-mortem interval” represents the main use of sarcosaprophagous beetles in forensic practice, while “Silphidae” may be a common emphasis at the intersection of forensic science and entomology research, indicating that these beetles may have a unique role in corpse decomposition and PMI estimation.

### 3.2. National and Regional Studies

**Figure 3:**
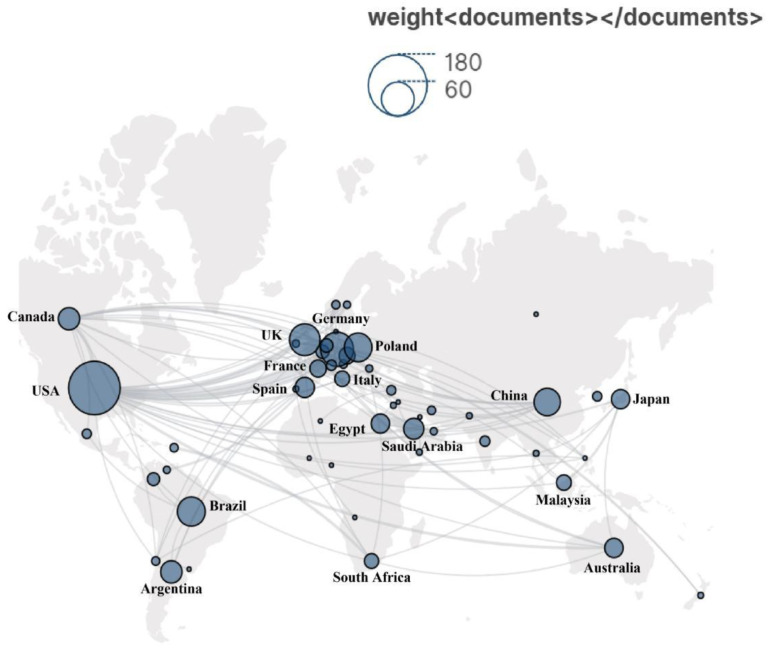
Country contributions and links between countries in the study of cadaver-associated beetles. The larger the number of publications from a country or region, the larger the size of nodes.

All the literature collected was distributed in 52 countries/regions (Figure 3). The USA has the highest number of publications, with a total of 156 articles. Germany follows with 64 articles, and the UK ranks third with 55 articles (Table 1). This reflects the leading position and important contribution of these countries to the field of sarcosaprophagous beetle research. In addition, North America and Europe cooperate frequently and show heightened academic activity.

### 3.3. Coleopteran Species on Cadavers

**Figure 4:**
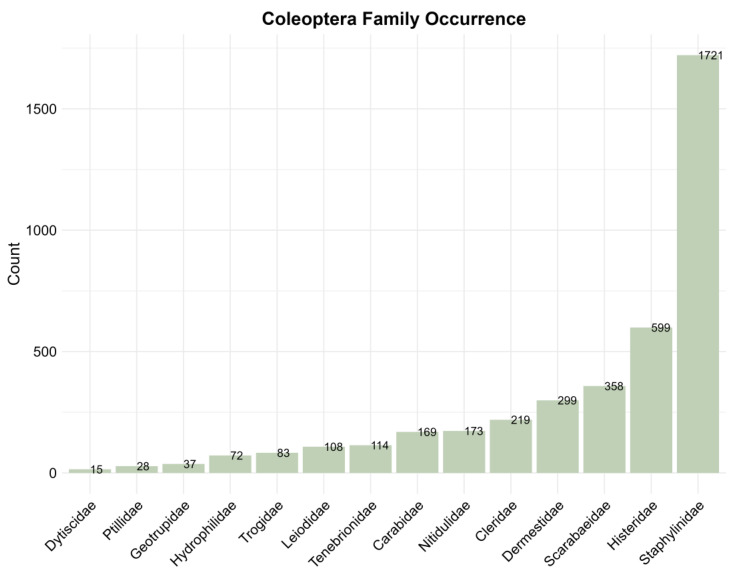
The occurrence frequency of cadaver-associated beetles in each family. The number of publications that record the species is first counted, and the results of the species statistics in the same family are added together, which is the Y-axis.

**Figure 5:**
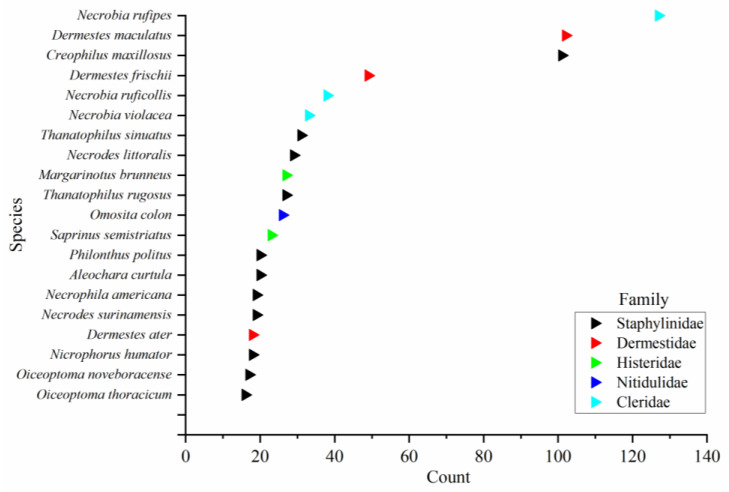
The top 20 most frequently recorded cadaver-associated beetles. The X axis represents the number of publications documenting the species.

There are many species of sarcosaprophagous beetles that play a complex and important role in the cadaver ecosystem. According to our statistics, a total of 1188 completely identified species of beetles from 68 families were collected. All beetles counted can be divided into two categories, one group was related to the carcass or carrion, which totaled 14 families and 1077 species (Supplementary Table S2), and the second group was considered to be accidental arrivals [26], which are mostly typical of phytophagous beetles and are recorded very infrequently, with a total of 54 families and 111 species (Supplementary Table S3). A specific description of the beetles in group two is not given here, as this study focuses on those associated with cadavers or carrion. The most frequently recorded family is Staphylinidae, far outnumbering the other families (Figure 4). In addition, we counted the top 20 most frequently recorded cadaver-associated beetles, among which *Necrobia rufipes* (Fabricius, 1781) (Coleoptera: Cleridae) were the most frequently recorded (Figure 5). As for the occurrence of species, we only provide the number of times that each family and species are recorded in the literature, which can roughly reflect the frequency of each species on cadavers worldwide. However, the number of publications varies from region to region, there are few or no reports of sarcosaprophagous beetles in some regions (even where sarcosaprophagous beetles are present), and there may be regional and habitat differences between species. Therefore, these factors may cause some bias between the statistical results and the real occurrence of species in a certain area. In the future, cadaver succession experiments can be carried out in different regions and environments to obtain more accurate region-specific species occurrence frequency.

### 3.4. Beetle Families Associated with Cadavers

#### 3.4.1. Staphylinidae

The Silphidae, also known as carrion beetles, consists of 187 species. However, it was recently reclassified as a subfamily Silphinae of Staphylinidae [27]. Here, we have a separate discussion of the subfamily Silphinae as their ecology and role on cadavers is different than that of most Staphylinidae. Silphinae beetles are 7 to 45 mm long, with diverse body shapes, all nearly round and flat, with the Palaearctic region as their origin and distribution center [28]. The Silphinae use vertebrate carcasses for reproduction and as a food source for themselves and their offspring [29]. Adults and larvae of the genera *Thanatophilus*, *Necrodes*, *Necrophila*, and *Oiceoptoma* are often observed on large cadavers, including humans, and are therefore considered of strong forensic relevance [30,31]. In particular, *Thanatophilus* species are present early in the decomposition of corpses and can begin reproducing within the first 24 h after death, characteristics that make them a very promising set of bioindicators to be used in the field of forensic entomology [32]. In addition, some of the genera *Nicrophorus* and *Ptomascopus* exhibit strong social behavior, nurturing and protecting their offspring. Although also found on large carcasses, the parents may often use small carcasses as a site for feeding, reproduction, and rearing their offspring during reproduction [8,33]. Silphinae beetles feed on the cadavers or preying on other species on the carcasses such as maggots. This suggests that they play a complex role in cadaver decomposition, acting as both decomposers and predators, and have a notable impact on energy cycling and material flow in the carcass ecosystem. A total of 74 species of Silphinae were counted, with *Thanatophilus sinuatus* (Fabricius, 1775) being the most frequently recorded (31 times), followed by *Necrodes littoralis* (Linnaeus, 1758) (29 times) and *Thanatophilus rugosus* (Linnaeus, 1758) (27 times) (Supplementary Table S2).

Disregarding Silphinae, the Staphylinidae also known as rove beetles, are one of the most diverse families of beetles, with about 67,000 described species worldwide [27]. Their diversity is not only reflected in the number of species, but also in their wide range of living environments and complex feeding habits [34]. Many species live in humid environments, such as coastal and waterside wetlands where they hide in dead leaves, bark, or decaying wood, where organic matter accumulates and attracts a variety of small animals and microorganisms, providing food for the beetles. Rove beetles act as decomposers or predators in the ecosystem, which is crucial for maintaining the ecological balance [35]. Most species of this group are predatory; those of forensic interest mainly feeding on the larvae of flies on the cadaver [36]. This behavior has a complex impact on the decomposition process, where they reduce the number of fly larvae and affect the structure of the insect community on cadavers, which can affect the decomposition speed of cadavers to a certain extent. Results show that 371 species of this group were found on carcasses, a far greater number of species than any others. *Creophilus maxillosus* (Linnaeus, 1758) is the species most abundant cadaver visitor of this family, with its occurrence far exceeding that of other species of this family (101 times) (Supplementary Table S2). *Creophilus maxillosus* frequently appeared on the cadavers of large vertebrates, where they reproduced and specialized in feeding on fly larvae, which is considered being of significant forensic value [37].

#### 3.4.2. Dermestidae

The Dermestidae are commonly known as skin beetles, and are a worldwide group of beetles that feed on a variety of products, from dried plant and animal tissues to stored grain and fiber [38]. They are major pests of stored products, causing significant economic losses. In addition, some species may occasionally act as intermediate hosts for parasites or vectors for disease and can cause allergic reactions [39]. Because this family prefers dry, protein-rich organic matter [40], they can accelerate the skeletonization process of animal carcasses to a certain extent, so in some cases, this family can be used to clean animal or human bone specimens to avoid bone damage [31,32,33,34,35,36,37,38,39,40,41,42,43]. The Dermestidae are regarded as valuable insect evidence in forensic investigations, especially the genus *Dermestes*, which are often found on dead bodies in the late stages of decomposition, where they usually feed on remains without organs and soft tissues [44]. We reviewed 34 cadaveric-related Dermestidae species, of which *Dermestes maculatus* DeGeer, 1774 was the most frequently recorded (102 times), followed by *D. frischi* Kugelman, 1792 (49 times) (Supplementary Table S2).

#### 3.4.3. Cleridae

These ‘checkered beetles’ comprise about 4000 species in the world, distributed in 320 genera, with the larvae and adults mainly feeding on other insects [45]. Some of the *Necrobia* species can also harm human-stored food, such as copra, cheese, dried fish, ham, and other protein-rich products [46], and are important storage pests, highlighting the adaptability of the family. There were only four species associated with carcasses, including *N. rufipes*, *N. ruficollis* (Fabricius, 1775), *N. violacea* (Linnaeus, 1758), and *Opetiopalpus sabulosus* Motschulsky, 1840. Of these, *N. rufipes* appeared the most frequently on cadavers (127 times), while the other three were only recorded 72 times (Supplementary Table S2).

#### 3.4.4. Histeridae

Clown beetles (Histeridae) are small beetles, usually no more than 10 mm long. There are 4252 species described, belonging to 391 genera, and they are particularly common in tropical and subtropical climates [47,48]. Due to their highly variable habitat preferences, they can be found in a variety of environments, including but not limited to feces, fungi, tree trunks, decaying fruit, roots, bird nests, and mammal or reptile burrows. In addition, they can live in decaying vegetation and carcasses [49]. This reflects the high level of adaptability and flexibility of the family in terms of food sources and living conditions. Adults and larvae are primarily predatory, feeding on mites and insects, and even exhibiting cannibalistic behavior when prey densities are low [50]. They are useful in forensic investigations, as they are attracted and present in large numbers in environments where cadavers are decomposing, especially when fly larvae are abundant on the cadaver [51,52]. These beetles are not only involved in the interaction between predators and prey on cadavers, but may also influence the rate and process of decomposition of cadavers by affecting the insect community structure on cadavers. Results from this study identify 146 species of this family found on carrion, of which *Margarinotus brunneus* (Fabricius, 1775) was recorded most frequently (27 times), followed by *Saprinus semistriatus* (L.G. Scriba, 1790) (23 times) (Supplementary Table S2).

#### 3.4.5. Nitidulidae

The Nitidulidae are known as sap beetles, and there are about 350 genera in the world, comprising more than 4500 species. These beetles are known for their complex diet, consuming flowers, plant sap, fungi, and fermenting and decaying plant and animal tissues [53]. The species are found in most countries of the world, reflecting their extensive adaptability and ecological diversity. The Nitidulidae cause significant economic impact by damaging stored grain, reducing grain quality, and providing favorable conditions for the growth of mold and fungi. In addition, their excrement and insect debris can contaminate grains and food, rendering them important storage pests [54]. A number of species with notable sarcosaprophagous tendencies, such as the genus *Omosita*, are of forensic interest. Of the 33 species counted, *O. colon* (Linnaeus, 1758) was most frequently recorded (26 times), followed by *O. discoidea* (Fabricius, 1775) (15 times) (Supplementary Table S2).

#### 3.4.6. Scarabaeidae

The Scarabaeidae family is almost universal, with nearly 4000 species recorded from all over the world. Although the vast majority of the species feed on plants or dung, studies show that some species, in particular the Scarabaeinae (known as true dung beetles) and the Aphodiinae (known as small dung beetles) were caught in large numbers in traps using carrion as bait [55,56]. However, there are different accounts as to whether these species are attracted directly to carrion or to the postmortem stomach contents. Midgley et al. [57] suggested that *Frankenbergerius forcipatus* (Harold, 1881) (Coleoptera: Scarabaeidae) may be more interested in the contents of the gastrointestinal tract exposed during decomposition than in the rotting flesh itself. Stone et al. [58] examined the distribution of dung beetles (Scarabaeinae and Aphodiinae) at the cranial and caudal ends of rat carcasses and found that they were attracted to the carrion itself. In this study, 154 species of scarabs were recorded from literature, of which *Onthophagus hecate* (Panzer, 1794) was recorded the most (nine times) (Supplementary Table S2).

#### 3.4.7. Geotrupidae

The Geotrupidae are commonly known as earth-boring dung beetles and are similar to the Scarabaeinae and Aphodiinae, with some morphological differences. There are more than 600 species worldwide, most of which live in arid regions. Adults and larvae generally feed on livestock droppings, carcasses, rotten wood, or saprophytic fungi [59]. In succession experiments, species of this family often appear late on the decaying cadavers. Here, 14 species were identified from cadavers, in particular *Anoplotrupes stercorosus* (Hartmann, 1791) (10 times) and *Geotrupes stercorarius* (Linnaeus, 1758) (seven times), which were recorded most frequently (Supplementary Table S2), showing a strong association with carrion.

#### 3.4.8. Trogidae

There are about 300 species of Trogid beetles worldwide, distributed in six extant genera [60]. These beetles are unique in their feeding habits, as both adults and larvae feed on various sources of keratin from animal nests and remains, including dry skin, feathers, hair, bones, dried feces, etc. [61]. Adults and larvae are usually found late in the skeletal decomposition of animal carcasses and are often considered being the last in a chain of decomposers to exploit carcasses. They can stay at a food source for a long time, sometimes completing several generations on the same carcass, as long as the carcass still provides them with adequate nutrition [62]. Of the 26 species that were recorded, *Omorgus suberosus* (Fabricius, 1775) was most abundant, appearing in the literature 16 times (Supplementary Table S2).

#### 3.4.9. Leiodidae

The Leiodidae are small beetles (1–7 mm) comprising 250 genera and 2000 species, and are considered as being generalist detritivores, feeding on decaying leaves, carrion and fungi, and often living in leaf litter and rock caves [63]. In particular, species of the Cholevinae subfamily, also known as “small carrion beetles” [64], are often present in cadaveric succession experiments and are considered of forensic importance. For the majority of Leiodidae, detailed data on biological characteristics and distribution are scarce, suggesting that there is still a lot of room for the scientific exploration of these beetles. Future research should focus on their ecological habits, distribution patterns, and role in the cadaveric environment. Among the 41 species counted here, *Sciodrepoides fumatus* (Spence, 1813) was recorded six times, followed by *Catops simplex* Say, 1825, and *C. basilaris* Say, 1823 (five times each) (Supplementary Table S2).

#### 3.4.10. Carabidae

The Carabidae, or ground beetles, are one of the largest beetle families, containing more than 40,000 species. Most are predatory and play an important role in biological balance and pest control in nature [65]. This family was not specifically studied in forensic entomology and was only documented in succession experiments [66]. Although 88 species were found on cadavers, suggesting high diversity, the frequency of occurrence of each species was low, and most species were recorded on cadavers only once (Supplementary Table S2). This suggests that the resources around the cadaver were attractive to these species, but that the attraction was not strong.

#### 3.4.11. Tenebrionidae

The Tenebrionidae family includes 2300 genera and 20,000 species worldwide [67]. Many of these species are detritivores, feeding on decaying organic matter, including dead plants and animals [68]. Some studies suggested that beetles in this family arrive at carcasses by accident [52,69], but the results of numerous succession experiments suggest that these species are attracted to the carcasses or the insects that surround them. Therefore, at least some Tenebrionids should be classified as forensic-related species. Of the 60 species counted, *Hylithus tentyroides* were most frequently associated with cadavers, although it was recorded only four times (Supplementary Table S2). Most of the other species were recorded only once or twice. This large diversity but low frequency on cadavers may be because, while carcasses are attractive to these beetles, they are not the key players in the decomposition process.

#### 3.4.12. Ptiliidae

The Ptiliidae are widely distributed, with nearly 1000 species in 100 genera worldwide. This family contains the smallest known beetles, less than 1 mm long [70]. Their small size allows them to live in decomposing wet organic matter and feed mainly on the fungi commonly found in fallen leaves, under vines, tree holes, dung, and lichens [69]. They are found on fish [71], rabbit [72], pig [73], human [74], and other carcasses, confirming the scavenging or sarcosaprophagous habits of the family. Although the number of Ptiliidae associated with cadavers is small (eight counted in this study, most of which were recorded only once (Supplementary Table S2)), they still have an important role to play in the decomposition of cadavers. It was suggested that their low frequency on cadavers is more likely because of the difficulty of collecting them due to their small size than to the absence of this group in entomological succession studies [69]. Thus, the presence of this group in entomological succession studies may be underestimated because of technical and methodological limitations, which need to be addressed in future studies.

#### 3.4.13. Water Beetles (Hydrophilidae and Dytiscidae)

The Hydrophilidae, also known as water scavenger beetles, is a large family of aquatic beetles distributed throughout the world. It currently includes 181 genera and more than 3100 species [75]. While most adults are herbivorous, feeding on aquatic plants, there are some species that are sarcosaprophagous, omnivorous, and occasionally prey on other insects or eat dung. Their larvae are mostly predators, and therefore many larvae could also be associated with the carcass and the sarcosaprophagous insects in the water. Most of the beetles are aquatic, but some species can live in specific environments on land, such as fresh feces, humus-rich soil, or decaying wet leaves [69]. This family is often found on land carcasses [76], buried carcasses [77], and water carcasses [78], feeding either directly on the carcass or on the eggs and larvae of other small insects on the carcass, such as Diptera. Twenty-two species of this family were recorded on cadavers, of which *Sphaeridium scarabaeoides* (Linnaeus, 1758) were mentioned the most (seven times) (Supplementary Table S2).

The Dytiscidae is the most diverse family of beetles inhabiting freshwater environments, with more than 4200 known species [79]. They are found in aquatic environments around the world, including ponds, lakes, and swamps, while a few live in the slow-flowing waters of streams and valleys [80]. They are carnivorous or saprophagous [81]. Although they are often found on water carcasses, little research attention is paid to this group, and many of the records only mention the genus or family. Only two recorded species were counted in this study (Supplementary Table S2).

The ability of the Hydrophilidae and Dytiscidae to survive on carcasses under water has potential forensic value. By feeding on the soft tissues, algae, or other microorganisms on the carcass, they affect decomposition, leaving recognizable patterns of damage or signs of biological activity, which can be used by forensic entomologists to analyze the decomposition stage of the body and to distinguish damage from ante-mortem injuries [81,82,83]. This study identified only a few beetles from these families associated with cadavers, possibly because most carcasses were found on land and there being too few experiments on cadaver succession in water. Conducting experiments on cadaver succession in water is complex and challenging compared to those on land, as the changes in water quality, flow velocity, water temperature, and other environmental factors can affect the decomposition process and insect activity. These factors are more difficult to control and simulate than the environmental factors of terrestrial environments [56,84]. This is also reflected in the inadequacy of aquatic insect identification and the limited number of taxonomic studies, which need to be addressed in future.

### 3.5. Identification of Immature Stages

Species identification is the first and important step in forensic entomological PMI estimation [85,86]. Insect development and succession are highly species-specific, and incorrect species identification may result in significantly deviated PMI estimations [87]. Insect are identified either using morphology or molecular methods. Morphology is the basis of insect species identification; however, taxonomic studies mostly focus on adult morphology, while forensic evidence mainly includes the immature stages (egg, larva, and pupa) of sarcosaprophagous insects [88]. Some studies tried to address this problem; for example, Williams et al. [89] described the morphology of the mature larvae of *Omosita nearctica* Kirejtshuk, 1987 and Diaz-Aranda et al. [90] provided key points for mature larval identification of 23 Coleopteran species in four different families (Cleridae, Dermestidae, Nitidulidae, and Staphylinidae). Unfortunately, these studies mainly focus on describing the final instar larvae, despite the fact that the cadaver usually plays host to larvae of all instars. In addition, for some sarcosaprophagous beetles, there is a great difference between the younger and the final instar larvae; for example, some species of the genera *Omosita* and *Nitidula* (Nitidulidae) show great variation in the color of the tergites of the larvae at different instars [91]. The description of the different instars is also conducive to age and, subsequently, PMI estimation. Therefore, it is necessary to identify and describe each of the different instars of sarcosaprophagous beetles.

Although molecular identification plays an important role in the identification of different species, the identification of different instars relies on morphological identification. Some scholars described three instars for some species of Silphinae, such as *T. rugosus* [92], *T. sinuatus* [32], and *Thanatophilus capensis* (Wiedemann, 1821) [87]. Hu et al. [91] also compared the morphological structure of three instar larvae of four Nitidulidae species using light microscopy and scanning electron microscopy and derived an identification key. Even so, there are few studies on the morphological identification of sarcosaprophagous beetle larvae, and even fewer studies on egg and pupal morphology, which need to be addressed to facilitate application in forensic entomology.

Molecular identification technology developed rapidly in recent years and became a routine method for insect species identification, playing an important role in quarantine, grain storage, and forensic entomology. The cytochrome oxidase subunit I (COI), cytochrome oxidase subunit II (COII), 16S ribosomal RNA (16SrRNA), 12S ribosomal RNA (12SrRNA), cytochrome b (Cytb), NADH dehydrogenase 5 (ND5), and other mitochondrial genes [19,93,94], as well as nuclear genes such as 18S ribosomal RNA (18SrRNA), 28S ribosomal RNA (28SrRNA), and internal transcribed spacer 2 (ITS2) are widely used in molecular identification of sarcosaprophagous Diptera [95,96]. In recent years, molecular identification studies on sarcosaprophagous beetles were carried out, such as by Cai et al. [97], who successfully identified six species of the superfamily Staphylinoidea using COI sequencing. Using COII as a genetic marker, Singh et al. [98] created a reference database for 11 species of four families of Coleoptera (Staphylinidae, Histeridae, Dermestidae, and Scarabaeidae) from two different states of India. Alajmi et al. [99] identified three beetle species collected from rabbit carcasses using 16SrRNA sequencing.

Studies show that the combination of multiple genes can achieve more accurate species identification than using a single gene sequence. Therefore, Su et al. [85] sequenced the 16SrRNA and Cytb genes of 23 Histeridae specimens collected from seven sites in six provinces in China, while Luo and Meng [11] identified the Silphinae of forensic significance in China based on COI and COII sequencing. However, compared with the amount of research on sarcosaprophagous flies, there is limited research on the molecular identification of sarcosaprophagous beetles. Future studies should focus on a wider range of sarcosaprophagous beetles, with targeted sampling and molecular identification of forensically important species that are currently lacking in reference sequences in online databases. In addition to traditional genetic markers, new molecular markers can also be identified and explored using the latest molecular technologies to provide new perspectives for species identification.

### 3.6. Age Estimation of Immature Stages

In forensic entomology, age estimation of the immature stages is based on morphological changes, including developmental duration, larval body length, and accumulated degree days. There are three frequently used developmental models that can easily estimate age: the isomorphen model, the isomegalen model, and the thermal summation model [100]. Much research was conducted on the development of flies, (especially the Calliphoridae, Sarcophagidae, and Muscidae) and in addition to studying the effect of temperature, diet [101,102], geographic factors [103,104], and toxicants [105,106] were also investigated. There are few studies on the development of sarcosaprophagous beetles, and the results of their review on these can be found in Table 2. Only a small fraction of the total number of sarcosaprophagous beetle species identified from the literature review have developmental studies associated with them. There is even less use of developmental data. Hu et al.’s study [107] showed that developmental data for only nine beetle species (*D. maculatus*, *D. frischii*, *D. ater*, *D. haemorrhoidalis*, *D. peruvianus*, *O. discicolle*, *T. micans*, *T. mutilatus*, and *C. maxillosus*) were applied to real cases. This is possibly because the rearing of beetles is more troublesome than flies, as the habits and environmental requirements of each beetle family are quite different, and it takes a long time to optimize the rearing protocols for different families or species.

Rearing protocols may affect the quality of the data and influence developmental patterns, especially when the insects are reared under conditions different from those found in nature. In most cases, the larvae of flies will aggregate on the carcass, so flies are raised in groups when doing development experiments in the laboratory. As beetles show large differences in habits between different families, some species of Staphylinidae, Dermestidae, and Nitidulidae larvae are gregarious; however, in some developmental experiments, they were reared individually [147,148]. To explore the effects of individual and collective rearing on development, Gruszka and Matuszewski [149] studied *N. littoralis* raised separately and in groups and found that group rearing was beneficial for development, resulting in lower mortality, shorter development times, and larger sizes, especially at low temperatures. This may be related to the fact that aggregation improves foraging efficiency and increases the temperature in the feeding microenvironment. Thus, to simulate the natural development process, it is recommended that beetles with gregarious habits be raised in clusters in developmental studies, which will not only improve the biological relevance of the study, but may also improve the accuracy and reliability of the experimental data.

Morphologically, the difference between beetles and flies is that the larvae have legs, more sclerotized structures and appendages, and more markings and patterns on the body surface [41]. Fly larvae have a degenerate head and no legs, and are maggot-like [150]. Other than body length and posterior spiracular changes, there are very few morphological indicators that can be used to determine instar age. In addition, the pupae of beetles are exarate pupa and can be easily observed from the outside [151], while the pupae of flies are coarctate pupa, and the insect body is difficult to observe through the opaque pupal shell [152]. Beetle larvae are thus richer in distinguishing morphological features, some of which include body length, head width, clypeus width, pronotum width, mesonotum width, labial palpomere length, urogomphi length, dorsal monocular distance, the distance between the urogomphi, ratio of different segments of labial palpomers, and length and ratio of different segments of urogomphi [32,91,92,153,154,155]. Discriminant analysis was even carried out for some morphological indicators. For example, Velásquez and Viloria used three morphometric indicators to distinguish the instar of *O. discicolle* well [153]; Frątczak and Matuszewski measured six morphological indicators of *N. littoralis* and *C. maxillosus*. Linear discriminant analysis found that almost all instars can be accurately classified, except for some newly molted larvae [155]. They also showed that fully sclerotized larvae require only a few indicators for accurate instar classification, whereas newly molted larvae will require more indicators [155]. Wang et al. [112] performed a cluster analysis on the head width and distance between the urogomphi of four instars of *N. ruficollis* and the total accuracy of discrimination was 84.44%. Similarly, Hu et al. [53] achieved a discriminatory accuracy of 99.74% for the cluster analysis of head width and the distance between the urogomphi for three instars of *N. rufipes*.

The gene expression patterns were also shown to up-regulate or down-regulate during development. The biggest advantage of differential gene expression technology is that the data are directly quantified by the instrument and can be analyzed for error, which agrees with the requirements of the Daubert rule of forensic science and is more likely to be accepted by court [156]. In addition, some studies emphasized that morphology is the basis for age estimation using differential gene expression, and that their combined use is more accurate than that of any single method [157,158]. The existing forensic entomological studies on differential gene expression mainly focus on flies, while research on sarcosaprophagous beetles is still lacking. Considering the multiple instars and diverse phenotypes of sarcosaprophagous beetles, it is important to identify differentially expressed genes that conform to the biological characteristics of their development. In recent years, with the rapid development of omics technology, transcriptomics and genomics have increasingly been applied to forensic entomology. As sequencing and data analysis technologies mature, transcriptome sequencing, gene screening, and bioinformatic analysis can potentially identify age-indicator genes for forensic entomological insect age estimation studies [159]. Other omics, such as the microbiome, proteome, and metabolome studies, are other novel methods for studying insect age and development and were successfully used in Dipteran studies [160].

### 3.7. Estimation of Arrival Time

The time elapsed since death until the insect reaches the cadaver is also called the pre-appearance interval (PAI) [161]. In blowflies, the PAI is very short under the right conditions, and they can reach the carcass and lay eggs in a few minutes. Therefore, the PMI estimations derived from blowfly offspring are infinitely close to the actual PMI without the need to incorporate PAI [88]. As sarcosaprophagous beetles arrive at the cadaver later, it is of great significance for accurate PMI estimation to determine the arrival time of sarcosaprophagous beetles.

Different species of insects react differently to decomposed cadavers. They arrive, reproduce, and eventually leave at different stages of cadaver decay according to their biological habits. This cyclical behavior of insects is called the pattern of community succession [162]. At first, studies simply described the insect succession in different locations, followed by the succession on cadavers in different seasons [163], different environments such as sunlight [164], indoors [165], inside a car [166], in water [78], and buried in the ground [167]. Meanwhile, studies also investigated succession patterns on different types of cadavers such as humans [74], pigs [168], rabbits [68], rats [169], and deer [170]. Of the many types of carcasses, pig carcasses are the most used, mainly because they are larger and more easily available, making them superior substitutes for human carcasses [171]. In addition, succession experiments were conducted on cadavers treated in different ways, such as hanging [172], burning [173], drugs [163], lime [174], and with or without clothing [175]. This resulted in a large amount of information on insect succession. Using these succession data, a succession matrix of the sarcosaprophagous insect community could be created. This model includes the arrival and departure time of each insect, and can depict the range of PMI, which is important for estimating the arrival time of sarcosaprophagous beetles.

Notably, the usefulness of species for the succession-based PMI estimation can be evaluated based on the species’ abundance on cadavers, length of the presence period, and regularity of appearance in the particular moment of decomposition. For example, Matuszewski et al. [176] evaluated different species on pig cadavers from different seasons in western Poland. It was found that *Saprinus planiusculus* Motschulsky, 1849 (Coleoptera: Histeridae), *S. semistriatus*, *N. littoralis*, and *C. maxillosus* had high usefulness for the succession-based PMI estimation. Of course, this usefulness may vary in different regions and habitats, so further research is needed.

Matuszewski [164], in his research on the arrival time of sarcosaprophagous beetles on carrion in Poland, reported that the PAI of *C. maxillosus* was mainly controlled by temperature, with an inverse correlation between temperature and PAI. Next, he verified 12 other sarcosaprophagous beetle species in the region and found that PAI was also highly correlated with temperature, so that the arrival time of sarcosaprophagous beetles can be estimated from temperature to provide a reliable estimate of PMI [177]. In a recent study, he verified the reliability of the established model for predicting the arrival time of sarcosaprophagous beetles, and the results show that the temperature–PAI model was more accurate in predicting PMI and had a wider range of application than the average seasonal and monthly PAI [178]. However, at present, this research is limited to the works of Matuszewski in Poland, and the applicability of this model to different regions and different species of beetles needs further research.

## 4. Conclusions

The Coleoptera are an important insect group sharing cadaver resources with flies and serve as a very important PMI indicator. By studying sarcosaprophagous beetles, forensic entomologists can enhance the accuracy of PMI estimation in the early and middle stages of cadaver decay, alongside flies as the indicator species in the initial stages. Additionally, their use can further improve and expand the time window of PMI estimation. Unfortunately, research on sarcosaprophagous beetles is limited, with no data available for many species, where even the number of species of cadaver-associated beetles is vague. In view of this, we reviewed studies and reports on the species of beetle that appeared on cadavers over the past 70 years, paying attention to the number of occurrences, larval species identification, instar identification, age estimation, and time of arrival. It is hoped that cadaver related beetles can receive more attention and play a greater role in forensic practice to achieve accurate PMI estimation by combining multiple methods.

## Figures and Tables

**Table 1:** Top 10 productive countries/regions based on the number of publications of cadaver-associated beetles.

Rank	Country	Publications	Total Citations	Average Citations
1	USA	156	5779	37.04
2	Germany	64	1808	28.25
3	UK	55	1216	22.11
4	Brazil	46	1072	23.30
5	Poland	45	1400	31.11
6	China	41	471	11.49
7	Argentina	27	533	19.74
8	Canada	27	850	31.48
9	Saudi Arabia	24	162	6.75
10	Spain	22	543	24.68

**Table 2:** Details of developmental studies on beetles associated with cadavers. The temperatures given in the table are the developmental temperatures of the beetles at different constant temperatures in lab conditions.

Family	Species	Temperature (°C)	Country	Reference
Carabidae	*Amara aenea*	17, 19, 22, 25, 28	Czech Republic	[108]
	*Amara familiaris*	17, 19, 22, 25, 28	Czech Republic	[108]
	*Amara fulvipes*	17, 19, 22, 25, 28	Czech Republic	[108]
	*Amara littorea*	17, 19, 22, 25, 28	Czech Republic	[108]
	*Amara ovata*	17, 19, 22, 25, 28	Czech Republic	[108]
	*Amara similata*	17, 19, 22, 25, 28	Czech Republic	[108]
	*Bembidion lampros*	5, 12, 17, 19, 22, 25, 30, 32	Denmark	[109]
	*Poecilus cupreus*	17, 20, 25	Germany	[110]
	*Pterostichus oblongopuncta*	7, 11, 14, 15, 17, 20, 25	Netherlands	[111]
Cleridae	*Necrobia ruficollis*	22, 25, 28, 31, 34	China	[112]
	*Necrobia rufipes*	22, 25, 28, 31, 34, 36	China	[113]
	*Necrobia rufipes*	22, 25, 28, 31	USA	[46]
Dermestidae	*Dermestes ater*	About18–22	India	[114]
	*Dermestes frischii*	19, 22, 25, 28, 31, 34	China	[41]
	*Dermestes frischii*	15, 20, 25, 30, 35	Spain	[39]
	*Dermestes haemorrhoidalis*	15, 20, 25, 30 or 32.5	England	[115]
	*Dermestes maculatus*	15, 20, 25, 30, 35	USA	[116]
	*Dermestes maculatus*	15, 20, 22, 24, 27, 30	Argentina	[117]
	*Dermestes maculatus*	20, 24, 28, 32	China	[118]
	*Dermestes maculatus*	15, 20, 25, 30, 35	Spain	[39]
	*Dermestes peruvianus*	15, 20, 25, 30 or 32.5	England	[115]
	*Dermestes tessellatocollis*	16, 19, 22, 25, 28, 31, 34	China	[119]
	*Dermestes undulatus*	15, 20, 25, 30, 35	Spain	[39]
Geotrupidae	*Geotrupes* *spiniger*	17–18	Germany	[120]
	*Geotrupes stercorosus*	17–18	Germany	[120]
	*Geotrupes vernalis*	17–18	Germany	[120]
Histeridae	*Euspilotus azureus*	10, 15, 20, 25, 30, 35	Brazil	[121]
Leiodidae	*Sciodrepoides watsoni*	12, 15, 18, 21, 28	Czech Republic	[122]
Nitidulidae	*Aethina tumida*	15, 20, 25, 30, 35, 37, 39	South Korea	[123]
	*Aethina tumida*	34, 24–28	USA	[124]
	*Carpophilus hemipterus*	20, 22.5, 25, 27.5, 30, 32.5, 35, 37.5, 40, 42.5	USA	[125]
	*Carpophilus humeralis*	20, 22.5, 25, 27.5, 30, 32.5, 35, 37.5, 40, 42.5	USA	[125]
	*Carpophilus marginellus*	18, 20, 25, 30	Japan	[126]
	*Carpophilus mutilatus*	20, 22.5, 25, 27.5, 30, 32.5, 35, 37.5, 40, 42.5	USA	[125]
	*Epuraea ocularis*	15, 20, 25, 30	Japan	[127]
	*Glischrochilus japonius*	20, 22.5, 25, 27.5, or 30	Japan	[128]
	*Glischrochilus quadrisignatus*	5, 10, 15, 17.5, 20, 25, 30, 35	USA	[129]
	*Nitidula rufipes*	16, 19, 22, 25, 28, 31, 34	China	[53]
	*Omosita colon*	16, 19, 22, 25, 28, 31	China	[130]
Scarabaeidae	*Bubas bison*	5, 15, 17.5, 20, 25, 30	Australia	[131]
	*Copris hispanus*	5, 15, 17.5, 20, 25, 30	Australia	[131]
	*Copris tripartitus*	15, 17.5, 20, 25, 27.5, 30	Korea	[132]
	*Digitonthophagus gazella*	10, 12, 14, 16, 18, 20, 22, 24, 26, 28, 30, 32	Canada	[133]
	*Onthophagous depressus*	25–27	USA	[134]
	*Onthophagus nuchicornis*	10, 12, 14, 16, 18, 20, 22, 24, 26, 28, 30, 32	Canada	[133]
	*Onthophagus taurus*	10, 12, 14, 16, 18, 20, 22, 24, 26, 28, 30, 32	Canada	[133]
	*Popillia japonica*	22, 26, 28, 30	USA	[135]
Staphylinidae	*Aleochara bilineata*	5, 9, 12, 17, 20, 24, 35	France	[136]
	*Aleochara bipustulata*	5, 9, 12, 17, 20, 24, 35	France	[136]
	*Creophilus maxillosus*	10, 12.5, 15, 17.5, 20, 22.5, 25, 27.5, 30, 32.5	Poland	[37]
	*Creophilus maxillosus*	17.5, 20.0, 22.5, 25.0, 27.5, 30.0, 32.5	China	[137]
	*Creophilus maxillosus*	16, 24, 32	USA	[138]
	*Creophilus maxillosus*	Room at about 24	USA	[139]
	*Necrodes littoralis*	14, 15, 16, 17, 18, 19, 20, 22, 26, 30	Poland	[13,140]
	*Necrophila brunnicollis*	18, 20, 22, 29	China	[141]
	*Oxelytrum discicolle*	15, 20, 28 and Outdoor (mean = 18.5)	Venezuela	[142]
	*Philonthus decorus*	10, 15, 20	Netherlands	[111]
	*Thanatophilus micans*	15, 17, 18, 19, 20, 25, 28.4, 35	South Africa	[21]
	*Thanatophilus micans*	15.0, 22.5, 32.5	South Africa	[143]
	*Thanatophilus mutilatus*	14.0, 15.0, 17.5, 19.0, 20.0, 22.5, 25.0, 27.5, 30.0	South Africa	[143]
	*Thanatophilus rugosus*	12, 14, 16, 18, 20, 22	Czech Republic	[144]
	*Thanatophilus sinuatus*	14, 16, 18, 20, 23, 26, 29	Poland	[145]
	*Thanatophilus sinuatus*	14, 16, 18, 20, 21, 24, 26	Czech Republic	[146]

## Data Availability

Data is provided within the manuscript or Supplementary Information files.

## Supplementary Materials

The following supporting information can be downloaded at: https://www.mdpi.com/article/10.3390/insects15090711/s1, Table S1: Keyword information for co-occurrence analysis of author keywords after disambiguation; Table S2: Coleoptera associated with cadavers recorded in various literature. A total of 14 families and 1077 species; Table S3: Coleoptera that arrived at cadavers by chance. A total of 54 families and 111 species. References [179,180,181,182,183,184,185,186,187,188,189,190,191,192,193,194,195,196,197,198,199,200,201,202,203,204,205,206,207,208,209,210,211,212,213,214,215,216,217,218,219,220,221,222,223,224,225,226,227,228,229,230,231,232,233,234,235,236,237,238,239,240,241,242,243,244,245,246,247,248,249,250,251,252,253,254,255,256,257,258,259,260,261,262,263,264,265,266,267,268,269,270,271,272,273,274,275,276,277,278,279,280,281,282,283,284,285,286,287,288,289,290,291,292,293,294,295,296,297,298,299,300,301,302,303,304,305,306,307,308,309,310,311,312,313,314,315,316,317,318,319,320,321,322,323,324,325,326,327,328,329,330,331,332,333,334,335,336,337,338,339,340] are cited in the supplementary materials
