# Supplementary material for: Research Status of Sarcosaprophagous Beetles as Forensic Indicators
Source: Insects. 2024 Sep 17;15(9):711. doi: 10.3390/insects15090711 (PMC11432003; doi:10.3390/insects15090711)
Supplement: Supplementary file 1 [file insects-15-00711-s001.zip › Table S1.pdf]

Table S1. Keyword information for co-occurrence analysis of author keywords after disambiguation. Cluster: The cluster is formed after the keywords are grouped by cluster analysis algorithm, 1, 2, 3 represent different clusters respectively. Average citations: The average number of citations of literature related to a particular keyword in a given period of time. Average normalized citations: The average number of citations to literature related to a particular keyword after normalization.

Keyword	Cluster	Occurrences	Average citations	Average normalized citations
arthropods	1	5	28.0	0.80
Calliphoridae	1	22	43.7	1.11
carrion decomposition	1	11	68.9	2.00
carrion insects	1	9	77.3	2.99
colonization	1	5	28.8	1.20
decomposition	1	55	34.9	1.16
decomposition stages	1	5	36.6	1.23
Dermestidae	1	21	12.5	0.77
development	1	10	15.4	2.03
entomology	1	10	46.4	0.81
forensic anthropology	1	6	14.7	0.72
forensic entomology	1	279	26.8	1.11
forensic sciences	1	49	34.8	0.98
forensic taphonomy	1	10	33.8	2.53
habitat	1	6	25.3	0.74
insects	1	13	30.1	1.27
instar determination	1	6	8.0	1.48
post-mortem interval	1	79	38.5	1.34
pre-appearance interval	1	8	21.8	1.13
Sarcophagidae	1	5	61.4	1.18
Staphylinidae	1	9	16.3	0.88
succession	1	86	43.6	1.34
taphonomy	1	7	20.9	1.25

biparental care	2	8	77.3	1.01
body size	2	6	26.5	0.56
carrion ecology	2	21	43.0	1.13
communal breeding	2	10	84.3	1.15
community ecology	2	6	5.7	0.83
competition	2	12	54.1	1.06
infanticide	2	7	27.7	0.71
juvenile hormone	2	11	29.9	0.66
parental care	2	44	25.7	0.97
phenotypic plasticity	2	5	23.4	1.09
reproduction	2	5	50.0	1.03
reproductive skew	2	5	29.0	0.65
reproductive success	2	5	41.8	0.74
resource availability	2	6	10.2	0.31
sexual conflict	2	5	35.0	0.95
Silphidae	2	156	25.1	0.85
terminal investment	2	5	45.0	1.22
biodiversity	3	10	14.2	0.86
carcass	3	40	35.7	1.06
carrion fauna	3	8	26.5	0.90
Coleoptera	3	68	25.3	0.93
Diptera	3	40	33.2	1.12
necrobiome	3	5	29.8	1.90
necrophagous insects	3	6	6.2	0.56
necrophagy	3	11	9.6	0.97
phoresy	3	6	19.0	0.61
seasonality	3	7	30.9	0.90
