# Supplementary material for: Research Status of Sarcosaprophagous Beetles as Forensic Indicators
Source: Insects. 2024 Sep 17;15(9):711. doi: 10.3390/insects15090711 (PMC11432003; doi:10.3390/insects15090711)
Supplement: Supplementary file 1 [file insects-15-00711-s001.zip › Table S2.pdf]

Table S2. Coleoptera associated with cadavers recorded in various literature. A total of 14 families and 1077 species. **Ref**: References where the species was first recorded. **N**: The number of publications documenting the species in this data set.

Family	Diet	Species	Ref	N	Types of cadaver or bait
Carabidae	Predaceous	*Abax ovalis*	[1]	1	rat
		Abax parallelepipedus	[1]	2	pig rat
		Abax sp.	[2]	1	pig
		Acinopus sp.	[2]	1	pig
		Acupalpus inornatus	[3]	1	pig
		Acupalpus sp.	[4]	2	pig bear
		Agonum cupreum	[5]	1	pig
		Agonum placidum	[5]	1	pig
		Agonum retractum	[5]	1	pig
		Agonum sp.	[5]	1	pig
		Amara obesa	[5]	1	pig
		Amara sinuosa	[6]	1	pig
		Amara sp.	[7]	1	wolf
		Amara torrida	[6]	1	pig
		Angionychus lividus	[5]	1	pig
		Anisodactylus verticalis	[5]	1	pig
		Argutoridius oblitus	[8]	1	pig
		Arthrostictus speciosus	[9]	1	pig
		Asaphidion curtum	[10]	1	pig
		Bembidion canadianum	[5]	1	pig
		Bembidion posticale	[8]	1	pig
		Bembidion quadrimaculatum	[5]	1	pig
		Bembidion sp.	[5]	2	pig
		Brachinus crepitans	[11]	1	pig
		Brachinus sp.	[12]	2	pig rat
		Bradycellus viduus	[8]	1	pig
		Calathus ingratus	[6]	1	pig
		Calathus micropterus	[13]	1	deer
		Calathus sp.	[14]	1	rabbit
		Calosoma scrutator	[4]	1	pig bear
		Calosoma trapezipenne	[15]	1	pig
		Carabus auronitens	[1]	1	rat
		Carabus chamissonis	[6]	1	pig
		Carabus coriaceus	[1]	2	pig rat
		Carabus hortensis	[1]	2	deer rat
		Carabus insulicola	[16]	1	rat
		Carabus jankowskii	[17]	1	pig
		Carabus linnaei	[1]	1	rat
		Carabus scheidleri	[1]	1	rat
		Carabus sp.	[18]	3	pig rabbit

Carabus ullrichii	[1]	1	rat
Carabus violaceus	[1]	2	deer rat
Carbonellia platensis	[8]	1	pig
Chlaenius erythropus	[4]	1	alligator
Chlaenius sp.	[5]	1	pig
Chlaenius variicornis	[3]	1	pig
Cicindela brevipilosa	[19]	1	pig
Cicindela sexguttata	[20]	1	pig bear deer alligator
Clivina sp.	[9]	1	pig
Cnemalobus curtisii	[15]	1	pig
Cnemalobus litoralis	[15]	1	pig
Cnemalobus sp.	[15]	2	pig
Cychrus attenuatus	[1]	1	rat
Dicaelus sculptilis	[5]	1	pig
Diplocheila striatopunctatus	[5]	1	pig
Dolichus halensis	[21]	1	pig
Dyschirius globosus	[7]	1	wolf
Dyscolus reflexicollis	[22]	1	rat
Dyscolus teter	[22]	1	rat
Epaphius secalis	[13]	1	deer
Galerita melanarthra	[12]	1	pig
Galerita sp.	[20]	3	pig deer
Harpalus aeneus	[23]	1	rabbit
Harpalus laticeps	[6]	1	pig
Harpalus nigritarsis	[6]	1	pig
Harpalus pensylvanicus	[5]	1	pig
Harpalus reversus	[24]	2	rabbit
Harpalus rubripes	[11]	1	pig
Harpalus rufipes	[25]	3	pig rabbit
Harpalus simplicidens	[26]	1	pig
Harpalus somnulentus	[6]	1	pig
Harpalus sp.	[5]	1	pig
Laemostenus sp.	[27]	1	human
Loricera pilicornis	[1]	1	rat
Loxandrus sp.	[28]	2	pig
Mesus sp.	[12]	1	pig
Molops piceus	[1]	1	rat
Notiobia sp.	[12]	1	pig
Notiophilus biguttatus	[23]	1	rabbit
Odontocheila fulgens	[29]	1	pig
Ophonus cribricollis	[2]	1	pig
Ophonus sp.	[2]	1	pig
Orthomus barbarus	[30]	2	pig
Oxypselaphus pusillus	[5]	1	pig

		Paecilus cupreus	[31]	1	pig
		Pelmatellus egenus	[8]	1	pig
		Pheropsophus javanus	[3]	1	pig
		Pheropsophus jessoensis	[32]	1	pig
		Pheropsophus sp.	[12]	1	pig
		Planetes puncticeps	[33]	2	pig
		Platynus assimilis	[34]	1	human
		Platynus decentis	[5]	1	pig
		Poecilus cupreus	[1]	2	rat fish
		Poecilus lucublanda	[5]	1	pig
		Poecilus versicolor	[1]	1	rat
		Pterostichus adstrictus	[6]	1	pig
		Pterostichus melanarius	[13]	1	deer pig
		Pterostichus niger	[25]	3	deer rabbit rat
		Pterostichus oblongopunctatus	[1]	2	deer rat
		Pterostichus pensylvanicus	[5]	1	pig
		Pterostichus pumilus	[35]	1	pig
		Pterostichus sp.	[18]	4	pig rabbit
		Scarites sp.	[12]	1	pig
		Selenophorus punctulatus	[8]	1	pig
		Stomis pumicatus	[1]	1	rat
		Syntomus americanus	[5]	1	pig
		Synuchus impunctatus	[5]	1	pig
		Tetragonoderus chalceus	[8]	1	pig
		Tetragonoderus laevigatus	[8]	1	pig
		Trechus quadristriatus	[2]	1	pig
		unidentified	[36]	34	human pig rabbit chicken bear deer alligator rat
Cleridae	Predaceous	*Necrobia rufipes*	[37]	127	human pig bear deer chicken rat cat impala rabbit goat bovine horse dog camel sheep wolf
		Necrobia ruficollis	[37]	38	human pig bear deer alligator cat rabbit
		Necrobia violacea	[38]	33	human pig rabbit rat deer wolf
		Necrobia sp.	[39]	10	human pig rabbit
		Opetiopalpus sabulosus	[40]	1	pig
		unidentified	[41]	10	human pig chicken dog
Dermestidae	Necrophagous	*Anthrenocerus australis*	[42]	1	human
		Anthrenus sp.	[43]	2	pig
		Anthrenus verbasci	[44]	1	human
		Attagenus obtusus	[45]	1	chicken
		Attagenus pellio	[46]	2	human deer
		Attagenus smirnovi	[44]	1	human
		Attagenus trifasciatus	[47]	1	pig
		Dermestes ater	[37]	18	human pig rat cat

		Dermestes bicolor	[48]	1	human
		Dermestes caninus	[49]	7	bear deer pig alligator gull rat
		Dermestes carnivorus	[50]	1	dog
		Dermestes coarctatus	[51]	4	rabbit deer pig
		Dermestes dimidiatus	[52]	3	pig
		Dermestes dimidiatus ab. rosea	[40]	1	pig
		Dermestes fasciatus	[5]	1	pig
		Dermestes frischii	[37]	49	human pig rabbit chicken rat cat squid dog camel goat wolf
		Dermestes haemorrhoidalis	[28]	3	human pig
		Dermestes hispanicus	[53]	1	squid
		Dermestes intermedius	[54]	1	pig
		Dermestes laniarius	[55]	7	fish squid pig
		Dermestes lardarius	[1]	8	human pig fish rat
		Dermestes leopardinus	[52]	1	pig
		Dermestes maculatus	[49]	102	human pig rabbit rat gull cat impala goat cattle horse sheep
		Dermestes marmoratus	[56]	1	pig rat
		Dermestes murinus	[1]	10	pig fish rat wolf
		Dermestes mustelinus	[53]	2	pig squid
		Dermestes olivieri	[54]	2	pig squid
		Dermestes pardalis	[53]	2	pig squid
		Dermestes peruvianus	[57]	4	human pig
		Dermestes sardous	[53]	3	pig squid
		Dermestes signatus	[5]	1	pig
		Dermestes talpinus	[38]	3	pig
		Dermestes tessellatocollis	[19]	6	human pig deer
		Dermestes sp.	[58]	18	human pig rat bovine rabbit wolf
		Dermestes undulatus	[23]	14	human pig rabbit dog fish squid
		Dermestes vorax	[59]	1	pig
		Trogoderma sp.	[60]	1	deer
		unidentified	[41]	14	human pig
Dytiscidae	Predaceous	*Acilius* sp.	[61]	1	pig
		Copelatus glyphicus	[4]	2	pig bear deer
		Copelatus sp.	[62]	2	pig
		Hydroglyphus sp.	[63]	2	pig rabbit
		Hypoporus sp.	[63]	2	pig rabbit
		Laccophilus sp.	[63]	2	pig rabbit
		Meridiorhantus validus	[64]	1	human
		Rhantus sp.	[65]	1	pig
		unidentified	[66]	2	pig
Geotrupidae	Coprophagous	*Anoplotrupes balyi*	[67]	1	pig
		Anoplotrupes hornii	[68]	1	pig
		Anoplotrupes stercorosus	[1]	10	human pig wolf rat

		Bolbapium striatopunctatum	[69]	1	pig
		Bolboceras falli	[5]	1	pig
		Chromogeotrupes auratus	[17]	1	pig
		Cnemotrupes semiopacus	[5]	2	pig bear deer alligator
		Geotrupes spiniger	[70]	1	pig
		Geotrupes splendidus	[4]	2	pig bear deer alligator
		Geotrupes stercorarius	[13]	7	human pig deer fish wolf
		Phelotrupes auratus	[60]	1	deer
		Phelotrupes laevistriatus	[16]	1	rat
		Trypocopris pyrenaeus	[71]	1	pig
		Trypocopris vernalis	[70]	5	pig wolf
		unidentified	[45]	2	human chicken
Histeridae	Saprophagous - Predaceous	*Abraeomorphus atomarius*	[37]	1	cat
		Abraeus perpusillus	[72]	1	pig
		Acritus nigricornis	[5]	1	pig
		Aeletes nicolasi	[73]	1	rabbit
		Aeletes sp.	[74]	1	rabbit
		Altholus depister	[75]	1	pig
		Altholus duodecimstriatus	[52]	3	pig
		Atholus americanus	[5]	1	pig
		Atholus corvinus	[72]	1	pig
		Atholus falli	[5]	1	pig
		Atholus rothkirchi	[37]	3	pig cat
		Atholus sedecimstriatus	[76]	1	pig
		Atholus sp.	[9]	1	pig
		Carcinops pumilio	[4]	14	human pig bear chicken fish
		Carcinops sp.	[77]	2	pig
		Carcinops troglodytes	[78]	1	pig
		Chaetabraeus bonzicus	[3]	1	pig
		Eremosaprinus sp.	[28]	1	pig
		Euspilotus aenicollis	[50]	1	dog
		Euspilotus assimilis	[4]	12	pig bear deer alligator
		Euspilotus azurenscens	[20]	1	pig bear deer alligator
		Euspilotus azureus	[79]	6	human pig
		Euspilotus bisignatus	[80]	1	pig
		Euspilotus caesopygus	[78]	2	pig
		Euspilotus conformis	[76]	1	pig
		Euspilotus connectens	[78]	3	pig
		Euspilotus eremita	[81]	1	cattle horse
		Euspilotus lacordairei	[82]	7	pig cattle horse
		Euspilotus lepidus	[78]	1	pig
		Euspilotus modestus	[82]	8	pig cattle horse rabbit
		Euspilotus niger	[8]	1	pig

Euspilotus nigrita	[74]	1	pig
Euspilotus ornatus	[82]	6	pig
Euspilotus parenthesis	[82]	5	pig cattle horse
Euspilotus patagonicus	[78]	5	pig cattle horse
Euspilotus pavidus	[82]	5	pig cattle horse
Euspilotus richteri	[78]	1	pig
Euspilotus simulatus	[20]	1	pig deer alligator
Euspilotus sp.	[57]	15	pig rat rabbit cattle horse
Euspilotus strobeli	[78]	1	pig
Geomysaprinus belioculus	[50]	1	dog
Geomysaprinus cheyennensis	[5]	1	pig
Gnathoncus buyssoni	[70]	2	pig
Gnathoncus nannetensis	[55]	4	pig fish
Gnathoncus nidorum	[70]	1	pig
Gnathoncus rotundatus	[52]	1	pig
Gnathoncus sp.	[83]	1	pig
Hister abbreviatus	[4]	8	pig bear deer alligator
Hister bipunctatus	[84]	1	rabbit
Hister cadaverinus	[25]	3	pig rabbit
Hister cavifrons	[85]	2	human pig
Hister coenosus	[86]	3	pig bear rat
Hister curvatus	[87]	1	pig
Hister depurator	[35]	2	pig bear deer alligator
Hister furtivus	[5]	4	pig
Hister grandicolis	[47]	1	pig
Hister helluo	[72]	1	pig
Hister illigeri	[52]	1	pig
Hister monitor	[88]	1	pig
Hister noma	[36]	2	pig
Hister punctifer	[50]	2	pig dog
Hister quadrinotatus	[54]	3	pig rabbit
Hister servus	[77]	1	pig
Hister simplicisternus	[3]	1	pig
Hister sp.	[58]	27	pig rabbit dog bovine chicken rodent wolf
Hister unicolor	[23]	8	pig rabbit rat fish
Holepta sp.	[89]	3	pig dog
Hololepta reichii	[78]	2	pig
Hypocaccus sp.	[88]	2	pig rat
Margarinotus ventralis	[23]	7	pig rabbit fish
Margarinotus binotatus	[30]	2	pig
Margarinotus bipustulatus	[72]	1	pig
Margarinotus brunneus	[5]	27	human pig fish deer
Margarinotus carbonarius	[1]	7	pig rat rabbit deer
Margarinotus faedatus	[68]	4	pig

Margarinotus harrisii	[5]	1	pig
Margarinotus hudsonicus	[5]	3	pig
Margarinotus immunis	[5]	1	pig
Margarinotus lecontei	[5]	3	pig
Margarinotus marginatus	[90]	3	pig
Margarinotus merdarius	[55]	3	pig fish
Margarinotus neglectus	[91]	2	pig deer
Margarinotus niponicus	[3]	1	pig
Margarinotus obscurus	[91]	1	pig
Margarinotus purpurascens	[54]	1	pig
Margarinotus reichardti	[3]	1	pig
Margarinotus ruficornis	[70]	5	pig
Margarinotus sp.	[92]	4	pig rat
Margarinotus striola	[1]	12	human pig rabbit rat fish
Margarinotus uncostriatus	[43]	2	pig
Margarinotus weymarni	[3]	1	pig
Margarinotus ignobilis	[93]	4	human pig
Merohister jekeli	[75]	7	human pig
Microsaprinus bonnairii	[94]	1	rabbit
Neopachylopus sp.	[62]	1	pig
Omalodes bifoveolatus	[79]	2	pig rabbit
Omalodes foveola	[79]	3	pig
Omalodes lucidus	[79]	1	pig
Omalodes planifrons	[9]	1	pig
Omalodes ruficlavis	[95]	1	pig
Omalodes sp.	[57]	4	pig dog rodent
Onthophilus globulosus	[30]	1	pig
Onthophilus punctatus	[72]	1	pig
Onthophilus striatus	[72]	2	pig
Operclipygus hospes	[96]	1	rabbit
Pachylister caffer	[37]	1	cat
Pachylister inaequalis	[54]	1	pig
Pachylopus fraternus	[49]	1	gull
Phelister sanguinipennis	[79]	1	pig
Phelister sp.	[97]	8	pig rabbit cattle horse rat
Phelister subrotundatus	[98]	2	pig
Phelister rufinotus	[78]	1	pig
Platysoma sp.	[88]	1	pig
Saprinus acuminatus	[47]	1	pig
Saprinus aeneus	[99]	7	human pig rabbit fish
Saprinus africanus	[100]	1	pig
Saprinus assimilis	[49]	1	gull
Saprinus azureus	[57]	1	pig
Saprinus bicolor	[101]	1	pig

Saprinus caerulescens	[54]	18	pig rabbit
Saprinus calatravensis	[52]	1	pig
Saprinus chalcites	[102]	13	human pig rabbit rat chicken
Saprinus conformis	[49]	1	gull
Saprinus cruciatus	[43]	3	pig
Saprinus cupreus	[103]	1	rabbit
Saprinus cyaneus	[103]	4	human pig rabbit
Saprinus detersus	[43]	5	pig
Saprinus diptychus	[104]	6	pig cattle horse
Saprinus distinguendus	[105]	1	pig
Saprinus externus	[52]	1	pig
Saprinus fimbriatus	[37]	1	cat
Saprinus furvus	[45]	6	human pig chicken squid
Saprinus georgicus	[43]	3	pig
Saprinus godet	[52]	1	pig
Saprinus immundus	[54]	1	pig
Saprinus laetus	[106]	1	unspecified
Saprinus lugens	[37]	9	pig cat
Saprinus maculatus	[45]	4	pig chicken
Saprinus magnoguttatus	[107]	1	human
Saprinus melas	[47]	3	pig
Saprinus moyses	[108]	6	rabbit
Saprinus niger	[45]	1	chicken
Saprinus niponicus	[3]	2	pig
Saprinus optabilis	[75]	1	pig
Saprinus oregonensis	[5]	1	pig
Saprinus pennsylvanicus	[109]	2	pig
Saprinus pharao	[43]	2	pig
Saprinus planiusculus	[55]	13	human pig fish
Saprinus politus	[45]	2	pig chicken
Saprinus prasinus	[54]	2	pig
Saprinus robustus	[54]	2	pig
Saprinus ruber	[108]	1	rabbit
Saprinus semistriatus	[25]	23	human pig rabbit goat dog camel wolf rat chicken fish
Saprinus splendens	[75]	12	pig human rat rabbit
Saprinus steppensis	[52]	1	pig
Saprinus strigil	[107]	1	pig
Saprinus subnitescens	[54]	8	pig
Saprinus subvirescens	[110]	1	unspecified
Saprinus tenuistriatus	[31]	4	pig squid
Saprinus sp.	[111]	26	human pig turtle impala rabbit wolf rat
Scapomegas auritus	[79]	1	pig
Xerosaprinus sp.	[22]	2	pig rat

		Xestipyge sp.	[89]	1	pig
		unidentified	[56]	39	human pig bear deer alligator bovine dog goat camel rat
Hydrophilidae	Predaceous	*Berosus* sp.	[65]	4	pig rabbit
		Cercyon convexiusculus	[55]	1	fish
		Cercyon floridanus	[4]	1	pig
		Cercyon haemorrhoidalis	[68]	2	pig
		Cercyon impressus	[55]	1	fish
		Cercyon lateralis	[25]	4	rabbit rat fish
		Cercyon melanocephalus	[55]	1	fish
		Cercyon minusculus	[68]	2	pig
		Cercyon quisquilius	[55]	1	fish
		Cercyon sp.	[5]	4	pig rat rabbit
		Cercyon tristis	[55]	1	fish
		Cercyon unipunctatu	[25]	2	rabbit fish
		Cercyon variegatus	[20]	1	alligator
		Cercyon versicolor	[20]	1	bear
		Cryptopleurum minutum	[37]	4	pig rat cat
		Dactylosternum sp.	[95]	1	pig
		Enochrus esuriens	[63]	2	pig rabbit
		Helochares nipponicus	[3]	1	pig
		Helochares sp.	[63]	3	pig rabbit
		Hydrochara sp.	[61]	2	pig
		Megasternini sp.	[79]	1	pig
		Megasternum obscurum	[1]	1	rat
		Megasternum posticatum	[38]	1	pig
		Pelosoma lafetertei	[112]	1	human
		Pelossoma sp.	[113]	1	rabbit
		Regimbartia attenuata	[63]	2	pig rabbit
		Sphaeridium bipustulatum	[35]	3	pig
		Sphaeridium lunatum	[38]	4	pig
		Sphaeridium scarabaeoides	[36]	7	human pig rabbit
		Sphaeridium sp.	[5]	2	pig
		Tropisternus sp.	[65]	1	pig
		unidentified	[41]	9	pig
Leiodidae	Saprophagous	*Agathidium confusum*	[1]	1	rat
		Agathidium mandibulare	[1]	1	rat
		Agathidium seminulum	[1]	1	rat
		Anisotoma humeralis	[1]	1	rat
		Anisotoma orbicularis	[1]	1	rat
		Apocatops nigrita	[114]	4	deer rat fish
		Catoposchema tasmaniae	[115]	1	pig
		Catops alpinus	[23]	1	rabbit
		Catops basilaris	[35]	5	pig

		Catops chrysomeloides	[23]	2	rabbit rat
		Catops coracinus	[1]	4	pig rat fish
		Catops fuliginosus	[23]	2	rabbit rat
		Catops fuscus	[25]	3	pig rabbit
		Catops grandicollis	[1]	3	pig rat
		Catops kirbii	[25]	4	pig rabbit rat
		Catops morio	[1]	3	human rat fish
		Catops nigricans	[1]	2	rat fish
		Catops picipes	[34]	1	human
		Catops simplex	[18]	5	pig rabbit deer alligator
		Catops sp.	[35]	1	pig
		Catops subfuscus	[1]	2	rat fish
		Catops tristis	[1]	3	human rat fish
		Catoptrichus frankenhaeuseri	[38]	1	pig
		Choleva sturmi	[1]	1	rat
		Choleva sp.	[84]	1	rabbit
		Colenis immunda	[1]	1	rat
		Colenis impunctata	[20]	1	pig bear deer
		Cortinicara gibbosa	[38]	1	pig
		Dissochaetus amazonicus	[79]	1	pig
		Dissochaetus murrayi	[116]	3	rat rabbit
		Dissochaetus sp.	[22]	2	rat rabbit
		Fissocatops westi	[1]	2	rat fish
		Hydnobius sp.	[97]	1	pig
		Leiodes sp.	[117]	1	pig
		Leptinus testaceus	[1]	1	rat
		Nargus velox	[70]	1	pig
		Pinodytes cryptophagoides	[35]	1	pig
		Prionochaeta opaca	[4]	4	pig bear deer alligator
		Pseudonemadus sp.	[118]	2	pig
		Ptomaphagus medius	[55]	1	fish
		Ptomaphagus sericatus	[23]	4	pig rabbit rat fish
		Ptomaphagus sp.	[22]	1	rat
		Ptomaphagus tenuicornis	[30]	2	pig
		Ptomaphagus subvillosus	[25]	2	rabbit rat
		Sciodrepoides alpestris	[1]	2	fish rat
		Sciodrepoides fumatus	[1]	6	pig rat fish
		Sciodrepoides terminans	[68]	2	pig
		Sciodrepoides watsoni	[1]	4	pig rat fish
		unidentified	[5]	8	human pig rabbit rat
Nitidulidae	Saprophagous	*Aethina villosa*	[22]	1	rat
		Amphotis marginata	[34]	1	human
		Brassicogethes aeneus	[13]	1	deer
		Carpophilus hemipterus	[119]	1	human

		Carpophilus marginellus	[3]	1	pig
		Carpophilus mutilatus	[37]	2	pig cat
		Carpophilus sp.	[38]	5	pig
		Conotelus sp.	[62]	1	pig
		Cychramus variegatus	[114]	1	deer
		Epurea marseuli	[1]	1	rat
		Epurea pygmaea	[1]	1	rat
		Epurea sp.	[1]	4	pig rat rabbit deer
		Epurea unicolor	[1]	1	rat
		Epurea variegata	[1]	1	rat
		Glischrochilus ipsodes	[33]	1	pig
		Glischrochilus quadripunctatus	[13]	3	pig deer
		Glischrochilus quadrisignatus	[68]	2	human pig
		Glischrochilus siepmanni	[68]	2	pig
		Haptoncus luteolus	[20]	1	alligator
		Nitidula bipunctata	[1]	9	human pig deer rat
		Nitidula carnaria	[54]	11	human pig squid
		Nitidula eremita	[120]	1	rabbit
		Nitidula flavomaculata	[121]	6	human pig
		Nitidula nigra	[122]	2	pig gull
		Nitidula punctatissima	[34]	1	human
		Nitidula rufipes	[23]	14	pig rabbit fish
		Nitidula sp.	[111]	5	pig turtle
		Nitidula ziczac	[5]	2	pig rat
		Omosita colon	[111]	26	human pig rabbit bear deer alligator rat fish turtle
		Omosita depressa	[1]	9	human pig rat fish
		Omosita discoidea	[23]	15	human pig rabbit rat fish
		Omosita japonica	[3]	1	pig
		Omosita nearctica	[123]	1	chicken
		Omosita sp.	[124]	6	human pig
		Pocadius ferrugineus	[23]	2	rabbit deer
		Stelidota geminata	[28]	2	pig gull
		Stelidota sp.	[95]	3	pig rat
		Urophorus humeralis	[24]	1	rabbit
		unidentified	[36]	25	human pig rabbit dog chicken
Ptiliidae	Saprophagous	*Acrotrichis dispar*	[55]	1	fish
		Acrotrichis grandicollis	[55]	1	fish
		Acrotrichis henrici	[55]	1	fish
		Acrotrichis intermedia	[55]	1	fish
		Acrotrichis rugulosa	[55]	1	fish
		Acrotrichis silvatica	[55]	1	fish
		Acrotrichis sp.	[38]	3	pig rabbit
		Actinopteryx sp.	[62]	1	pig

		Nephanes sp.	[4]	2	pig deer alligator
		Ptenidium nitidum	[125]	1	deer
		Pteryx sp.	[18]	1	rabbit
		Ptiliola kunzei	[55]	1	fish
		unidentified	[37]	13	human pig chicken rat rabbit cat
Scarabaeidae	Omnivorous	*Acrossidius tasmaniae*	[103]	1	rabbit
		Acrossus depressus	[114]	2	deer rat
		Acrossus luridus	[114]	1	deer
		Acrossus rufipes	[114]	3	pig deer
		Agoliinus guttatus	[68]	2	pig
		Ammoecius brevis	[114]	1	deer
		Anachalcos convexus	[126]	1	antelope
		Anomala aulacoides	[26]	1	pig
		Anomala innuba	[4]	1	deer
		Aphodius fimetarius	[55]	4	pig fish
		Aphodius lividus	[37]	2	pig cat
		Aphodius sp.	[45]	6	pig wolf chicken
		Archophileurus chaconus	[8]	1	pig
		Archophileurus fodiens	[8]	1	pig
		Ataenius aequalis	[69]	1	pig
		Ataenius confertus	[127]	1	pig
		Ataenius pacificus	[37]	1	cat
		Ataenius picinus	[97]	2	pig
		Ataenius platensis	[20]	2	pig deer alligator
		Ataenius sp.	[69]	4	pig rabbit
		Ataenius spretulus	[128]	1	rat
		Ataenius strigatus	[128]	1	rat
		Ateuchus carbonarius	[9]	3	pig
		Ateuchus histeroides	[20]	3	pig rat alligator
		Ateuchus sp.	[77]	1	pig
		Ateuchus vividus	[69]	1	pig
		Bodilopsis rufus	[70]	1	pig
		Calamosternus granarius	[68]	4	pig
		Canthidium cavifrons	[129]	1	pig
		Canthidium depressum	[79]	1	pig
		Canthidium dispar	[129]	1	pig
		Canthidium humerale	[130]	1	beef
		Canthidium hyla	[131]	1	pig
		Canthidium manni	[130]	3	pig beef
		Canthidium moestum	[129]	1	pig
		Canthidium sp.	[116]	4	pig rat beef
		Canthon bispinus	[29]	1	pig
		Canthon carbonarius	[130]	1	beef
		Canthon cyanellus	[77]	1	pig

Canthon fuscipes	[132]	1	rat
Canthon lividus	[129]	1	pig
Canthon luctuosus	[129]	1	pig
Canthon maldonadoi	[130]	1	beef
Canthon mutabilis	[130]	2	human beef
Canthon oliverioi	[129]	1	pig
Canthon ornatus	[8]	1	pig
Canthon pilularius	[128]	1	rat
Canthon sordidus	[79]	1	pig
Canthon sp.	[133]	5	pig beef
Canthon triangularis	[79]	1	pig
Canthon virens	[69]	3	pig beef
Canthon viridis	[128]	1	rat
Chilothorax distinctus	[68]	5	pig
Cinacanthus militaris	[8]	1	pig
Coenochilus ventricosus	[88]	1	pig
Colobopterus erraticus	[68]	3	pig rat
Copris fricator	[128]	1	rat
Copris minutus	[128]	2	pig bear deer alligator rat
Copris sp.	[134]	2	pig
Coprophanaeus cyanescens	[9]	2	pig
Coprophanaeus ensifer	[133]	3	pig
Coprophanaeus horus	[131]	1	pig
Coprophanaeus jasius	[130]	2	beef
Coprophanaeus lancifer	[135]	2	pig
Coprophanaeus milon	[29]	1	pig
Coprophanaeus pertyi	[130]	3	pig beef
Coprophanaeus pluto	[77]	1	pig
Coprophanaeus saphirinus	[129]	1	pig
Coprophanaeus sp.	[58]	3	pig
Cotinis nitida	[136]	1	pig
Degallieridium sp.	[8]	1	pig
Deltochilum brasiliense	[133]	2	pig
Deltochilum cristatum	[129]	1	pig
Deltochilum gibbosum	[4]	2	pig bear deer
Deltochilum guyanense	[79]	1	pig
Deltochilum icarus	[79]	2	pig
Deltochilum irroratum	[130]	1	beef
Deltochilum peruanum	[79]	1	pig
Deltochilum verruciferum	[130]	3	pig beef
Deltochilum sp.	[137]	4	pig rat
Diabroctis mimas	[130]	2	pig beef
Dichelonyx sp.	[5]	1	pig
Dichotomius amplicollis	[138]	2	pig

Dichotomius assifer	[113]	1	rabbit
Dichotomius bicuspis	[129]	2	pig
Dichotomius boreus	[79]	1	pig
Dichotomius bos	[130]	2	pig beef
Dichotomius geminatus	[9]	2	pig
Dichotomius nisus	[130]	7	pig beef
Dichotomius opacipennis	[69]	1	pig
Dichotomius semisquamosus	[130]	1	beef
Dichotomius sericeus	[130]	1	beef
Dichotomius sp.	[97]	5	pig rat
Digitonthophagus gazella	[130]	2	beef
Diloboderus abderus	[29]	1	pig
Dyscinetus dubius	[12]	1	pig
Dyscinetus sp.	[97]	1	pig
Euphoria sepulcralis	[127]	1	pig
Eupleurus subterraneus	[68]	2	pig
Eurysternus aeneus	[69]	1	pig
Eurysternus hirtellus	[130]	1	beef
Eurysternus hypocrita	[79]	1	pig
Eurysternus parallelus	[133]	1	pig
Eurysternus velutinus	[137]	1	rat
Eurysternus sp.	[116]	3	pig rat
Frankenbergerius forcipatus	[139]	1	dog
Gymnopleurus sp.	[88]	2	pig
Hoplia sp.	[127]	1	pig
Labarrus lividus	[128]	1	rat
Labarrus pseudolividus	[69]	4	pig rat
Lechorodius terminalis	[128]	1	rat
Leucothyreus sp.	[97]	1	pig
Limarus maculatus	[114]	1	deer
Maladera insanabilis	[140]	4	pig rabbit rat
Maladera sp.	[141]	1	pig
Malagoniella astyanax	[130]	1	beef
Megathopa sp.	[116]	1	rat
Melinopterus femoralis	[128]	1	rat
Melinopterus prodromus	[68]	3	pig
Melinopterus sphacelatus	[114]	1	deer
Mimela splendens	[33]	1	pig
Nimbus contaminatus	[114]	3	deer wolf
Ontherus appendiculatus	[69]	2	pig
Ontherus digitatus	[130]	2	pig beef
Ontherus sp.	[130]	3	pig beef
Ontherus sulcator	[29]	2	pig
Onthophagus binodis	[142]	1	pig

Onthophagus buculus	[57]	2	pig
Onthophagus coenobita	[70]	1	pig
Onthophagus crassicollis	[143]	2	pig sheep
Onthophagus cyanellus	[22]	1	rat
Onthophagus fodiens	[144]	2	pig
Onthophagus hecate	[111]	9	pig bear deer turtle rat
Onthophagus hirculus	[130]	3	pig beef
Onthophagus incensus	[37]	1	cat
Onthophagus joannae	[7]	1	wolf
Onthophagus knausi	[128]	1	rat
Onthophagus lenzii	[3]	2	pig
Onthophagus nitidulus	[140]	3	rabbit
Onthophagus nuchicornis	[38]	6	pig fish
Onthophagus opacicollis	[30]	1	pig
Onthophagus orpheus pseudopheus	[4]	2	bear rat
Onthophagus ovatus	[55]	2	pig fish
Onthophagus pennsylvanicus	[145]	3	pig rat
Onthophagus pexatus	[103]	1	pig
Onthophagus proletarius	[75]	2	human pig
Onthophagus similis	[11]	1	pig
Onthophagus sp.	[146]	16	pig impala rabbit wolf beef
Onthophagus striatulus	[20]	1	deer
Onthophagus taurinus	[147]	4	human pig
Onthophagus taurus	[145]	1	pig
Onthophagus variegatus	[148]	1	rabbit
Oscarinus rusicola	[128]	3	rat deer
Parataenius derbesis	[29]	1	pig
Phanaeus saphirinus	[116]	1	rat
Phanaeus sp.	[105]	2	pig
Phanaeus vindex	[145]	2	pig rat
Phyllophaga bilobatata	[4]	1	pig
Planolinus fasciatus	[114]	1	deer
Popillia mutans	[33]	1	pig
Pseudagolius bicolor	[128]	2	pig bear deer rat alligator
Pseudataenius socialis	[128]	1	rat
Pseudocanthon perplexus	[20]	2	pig deer alligator rat
Pseudocanthon sp.	[8]	1	pig
Rhyssemus sulcatus	[149]	1	human
Saprosites pygmaeus	[37]	1	cat
Sarophorus tuberculatus	[149]	1	human
Scarabaeus rugosus	[21]	1	pig
Scybalocanthon nigriceps	[150]	1	frog
Scybalocanthon sp.	[133]	1	pig
Serica brunnea	[114]	1	deer

		Sulcophaneus menelas	[131]	1	pig
		Tetraechma balteata	[132]	1	rat
		Tetraechma liturata	[130]	1	beef
		Teuchestes fossor	[114]	2	deer
		Trichillum externepunctatum	[69]	3	pig
		Trichillum sp.	[130]	3	pig beef
		Trochalus crampelanus	[88]	1	pig
		Uroxys sp.	[22]	3	pig rat
		Volinus sticticus	[114]	2	pig deer
		unidentified	[36]	23	human pig chicken rat
Staphylinidae (Silphinae)	Necrophagous - Predaceous	*Aclypea daurica*	[151]	1	unspecified
		Dendroxena quadrimaculata	[152]	1	pig
		Diamesus osculans	[75]	6	human pig
		Eusilpha bicolor	[33]	2	pig rabbit
		Heterosilpha ramosa	[38]	5	pig
		Necrodes littoralis	[153]	29	human pig rat deer wolf fish squid
		Necrodes nigricornis	[154]	4	pig
		Necrodes sp.	[124]	1	pig
		Necrodes surinamensis	[38]	19	human pig bear deer alligator rat bovine raven
		Necrophila americana	[155]	19	human pig deer alligator rat bovine
		Necrophila brunnicollis	[156]	6	pig
		Necrophila cyaniventris	[39]	1	rabbit
		Necrophila jakowlewi	[154]	3	pig deer
		Necrophila japonica	[16]	1	rat
		Necrophila sp.	[157]	1	human
		Necrophilus hydrophiloides	[27]	1	human
		Necrophilus maculifrons	[17]	3	pig
		Nicrophorus antennatus	[52]	1	pig
		Nicrophorus basalis	[144]	1	pig
		Nicrophorus carolinus	[158]	1	rat
		Nicrophorus concolor	[21]	5	pig rat deer
		Nicrophorus defodiens	[35]	4	pig
		Nicrophorus germanicus	[52]	1	pig
		Nicrophorus humator	[25]	18	human pig rabbit rat fish squid
		Nicrophorus hybridus	[5]	2	pig
		Nicrophorus interruptus	[55]	7	human pig rabbit rat wolf fish squid
		Nicrophorus inverstigator	[35]	11	human pig rabbit fish
		Nicrophorus nepalensis	[151]	1	unspecified
		Nicrophorus nigritus	[27]	1	human
		Nicrophorus obscurus	[5]	1	pig
		Nicrophorus olidus	[138]	1	pig
		Nicrophorus orbicollis	[159]	8	human pig rat beef

Nicrophorus przewalskii	[151]	1	unspecified
Nicrophorus pustulatus	[158]	6	human pig rat
Nicrophorus quadripunctatus	[154]	3	pig rat
Nicrophorus sayi	[111]	4	pig turtle
Nicrophorus sepultor	[55]	1	fish
Nicrophorus sinensis	[151]	1	unspecified
Nicrophorus sp.	[35]	10	human pig
Nicrophorus tomentosus	[49]	11	human pig rabbit rat gull beef
Nicrophorus vespillo	[25]	10	pig rabbit rat deer fish squid
Nicrophorus vespilloides	[153]	15	human pig deer rat rabbit wolf fish
Nicrophorus vestigator	[55]	3	pig fish squid
Nicrophorus marginatus	[111]	7	pig rabbit rat beef turtle
Oiceoptoma inaequale	[4]	6	pig bear deer alligator rat beef
Oiceoptoma noveboracense	[49]	17	human pig rat beef gull turtle
Oiceoptoma rugulosum	[20]	2	pig bear deer
Oiceoptoma sp.	[92]	2	human pig
Oiceoptoma subrufum	[151]	1	unspecified
Oiceoptoma thoracicum	[23]	16	pig rabbit deer rat wolf fish
Oxelytrum apicale	[160]	1	pig
Oxelytrum cayennense	[79]	2	pig
Oxelytrum discicolle	[116]	15	human pig rat rabbit
Oxelytrum erythrurum	[112]	1	human
Oxelytrum lineatocolle	[161]	1	human
Oxelytrum sp.	[58]	5	pig rabbit
Ptomaphila lacrymosa	[118]	7	pig rat
Ptomaphila perlata	[162]	2	pig
Ptomascopus morio	[21]	4	rabbit rat deer
Ptomascopus plagiatus	[26]	3	pig
Silpha atrata	[1]	2	pig rat
Silpha carinata	[55]	2	rabbit fish
Silpha obscura	[55]	2	pig fish
Silpha obscura orientalis	[54]	1	pig
Silpha perforata	[151]	1	unspecified
Silpha puncticollis	[43]	2	pig
Silpha sp.	[11]	2	pig
Silpha tristis	[55]	4	pig deer rabbit fish
Thanatophilus coloradensis	[18]	3	human pig rabbit
Thanatophilus ferrugatus	[54]	2	pig
Thanatophilus lapponicus	[35]	12	pig rabbit rat
Thanatophilus micans	[141]	5	human pig sheep
Thanatophilus mutilatus	[149]	1	human
Thanatophilus pilosus	[151]	1	unspecified
Thanatophilus roborowskyi	[151]	1	unspecified
Thanatophilus ruficornis	[43]	7	pig deer squid

		Thanatophilus rugosus	[25]	27	pig rabbit rat wolf squid fish
		Thanatophilus sagax	[6]	1	pig
		Thanatophilus sinuatus	[25]	31	human pig rabbit rat wolf fish squid
		Thanatophilus sp.	[146]	4	pig impala
		Thanatophilus truncatus	[128]	1	rat
		unidentified	[159]	9	human pig chicken
Staphylinidae (Rove beetles)	Predaceous	*Achenomorphus corticinus*	[155]	1	rat
		Acidota cruentata	[1]	2	pig rat
		Acrolocha diffusa	[68]	2	pig
		Acrotona aterrima	[70]	3	pig human deer
		Acrotona muscorum	[70]	1	pig
		Acrotona parvula	[163]	2	pig
		Acylophorus sp.	[28]	2	pig
		Agelosus weisei	[33]	2	pig deer
		Aleochara bilineata	[164]	1	pig
		Aleochara bimaculata	[68]	2	pig
		Aleochara bipustulata	[70]	7	pig deer rat
		Aleochara bonariensis	[28]	1	pig
		Aleochara brachialis	[118]	2	pig
		Aleochara brevipennis	[52]	2	pig
		Aleochara caviceps	[22]	1	rat
		Aleochara clavicornis	[165]	2	pig
		Aleochara cuniculorum	[30]	2	pig
		Aleochara curtula	[25]	20	human pig rabbit rat goat
		Aleochara fumata	[68]	2	pig
		Aleochara funebris	[163]	2	pig
		Aleochara haematoptera	[52]	1	pig
		Aleochara haemorrhoidalis	[83]	1	pig
		Aleochara intricate	[54]	6	pig
		Aleochara laevigata	[52]	1	pig
		Aleochara lanuginosa	[125]	1	deer
		Aleochara lata	[49]	11	human pig bear deer alligator gull rat
		Aleochara lateralis	[57]	1	pig
		Aleochara lustrica	[4]	2	pig bear deer alligator
		Aleochara maculata	[52]	1	pig
		Aleochara notula	[155]	3	pig rat
		Aleochara oxypodia	[77]	1	pig
		Aleochara pacifica	[21]	1	rabbit
		Aleochara pseudochrysorrhoa	[74]	3	human rabbit
		Aleochara puberula	[26]	2	pig
		Aleochara ruficornis	[34]	1	human
		Aleochara sculptiventris	[68]	2	pig
		Aleochara sekanai	[68]	2	pig

Aleochara sp.	[166]	18	pig impala rat rabbit
Aleochara spadicea	[52]	1	pig
Aleochara speculifera	[83]	1	pig
Aleochara stichai	[164]	3	pig
Aleochara tristis	[43]	2	pig
Aleochara verna	[155]	6	human pig rat
Althetini sp.	[38]	1	pig
Ambodina sp.	[167]	2	pig
Amischa soror	[34]	1	human
Anacyptus sp.	[62]	1	pig
Anotylus complanatus	[43]	2	pig
Anotylus insignitus	[166]	2	pig
Anotylus inustus	[43]	7	pig
Anotylus laqueatus	[70]	1	pig
Anotylus mutator	[163]	2	pig
Anotylus pumilus	[43]	2	pig
Anotylus rugosus	[11]	3	pig rat
Anotylus sculpturatus	[70]	11	human pig deer bear rabbit alligator
Anotylus sp.	[155]	13	pig rat bear deer rabbit
Anotylus tetracarinatus	[1]	3	pig rat deer
Anotylus vinsoni	[37]	1	cat
Anthobium atrocephalum	[70]	3	pig
Anthobium melanocephalum	[114]	1	deer
Anthobium unicolor	[23]	1	rabbit
Arpedium quadrum	[114]	1	deer
Atheta aeneicollis	[30]	2	pig
Atheta atramentaria	[125]	1	deer
Atheta boreella	[163]	2	pig
Atheta britanniae	[70]	3	pig
Atheta cadaverina	[70]	3	pig
Atheta campbelli	[68]	1	pig
Atheta canescens	[168]	1	pig
Atheta castanoptera	[163]	2	pig
Atheta celata	[168]	1	pig
Atheta cinnamoptera	[163]	5	human pig
Atheta corvina	[1]	2	pig rat
Atheta crassicornis	[1]	3	pig rat
Atheta dadopora	[70]	3	pig
Atheta districta	[68]	2	pig
Atheta divisa	[1]	2	pig rat
Atheta excellens	[114]	1	deer
Atheta gagatina	[168]	2	pig
Atheta hansseni	[163]	2	pig
Atheta harwoodi	[52]	1	pig

Atheta iheringi	[74]	2	rabbit
Atheta incognita	[52]	1	pig
Atheta intermedia	[163]	1	pig
Atheta laevana	[70]	3	pig
Atheta laticollis	[43]	1	pig
Atheta longicornis	[70]	5	pig deer
Atheta marcida	[52]	1	pig
Atheta monticola	[163]	2	pig
Atheta nigra	[70]	1	pig
Atheta obscuripennis	[80]	1	pig
Atheta occulta	[52]	1	pig
Atheta paracrassicornis	[163]	2	pig
Atheta parvicornis	[43]	2	pig
Atheta picipes	[163]	2	pig
Atheta pseudcrenuliventris	[68]	2	pig
Atheta ravilla	[70]	3	pig
Atheta savardae	[68]	2	pig
Atheta smetanai	[68]	2	pig
Atheta sodalis	[43]	4	pig
Atheta sordidula	[70]	1	pig
Atheta subtilis	[1]	1	rat
Atheta testaceipes	[54]	1	pig
Atheta triangulum	[125]	1	deer
Atheta trinotata	[1]	1	rat
Atheta vaga	[1]	1	rat
Atheta sp.	[13]	11	human pig deer rat
Atrecus affinis	[114]	1	deer
Aulacocypus gloriosus	[26]	1	pig
Autalia longicornis	[70]	3	pig
Autalia rivularis	[70]	1	pig
Belonuchus rufipennis	[20]	4	pig bear deer alligator rat bovine
Belonuchus sp.	[169]	5	pig rabbit rat
Bisnius blandus	[68]	3	human pig
Bisnius cephalotes	[164]	5	human pig deer fish
Bisnius fimetarius	[165]	11	pig rat deer fish
Bisnius inquietus	[155]	1	rat
Bisnius nitidulus	[170]	2	pig
Bisnius parcus	[170]	2	pig
Bisnius pseudoparcus	[163]	2	pig
Bisnius puella	[55]	1	fish
Bisnius sordidus	[54]	4	human pig deer fish
Bobitobus lunulatus	[168]	2	pig
Bolitochara bella	[1]	1	rat
Bolitochara obliqua	[1]	2	pig rat

Bolitochara pulchra	[164]	1	pig
Bolitochara tecta	[163]	1	pig
Brachygluta fossulata	[1]	1	rat
Bryophacis maklini	[43]	2	pig
Cafius seminitens	[21]	1	rabbit
Carpelimus corticinus	[93]	1	human
Carpelimus sp.	[8]	2	pig
Cephennium majus	[1]	1	rat
Coprophilus striatulus	[34]	1	human
Coproporus hepaticus	[22]	1	rat
Coproporus sp.	[113]	2	rabbit bovine
Cordalia obscura	[43]	2	pig
Creochara brevipennis	[19]	1	pig
Creophilus erythrocephalus	[118]	9	human pig rat rabbit
Creophilus lanio	[171]	2	human pig
Creophilus maxillosus	[25]	101	human pig rabbit bear deer alligator rat gull cat bovine wolf
Creophilus ocufatus	[172]	1	pig
Creophilus sp.	[157]	2	pig rabbit
Creophilus variegatus	[112]	1	human
Cryptobium sp.	[69]	2	pig
Cyparium sp.	[127]	1	pig
Dalotia coriaria	[93]	1	human
Deinopteroloma subcostatum	[38]	1	pig
Dianous sp.	[62]	2	pig
Dibelonetes hybridus	[69]	1	pig
Diestota sp.	[22]	1	rat
Dinothenarus badipes	[159]	2	pig
Dinothenarus capitatus	[68]	4	pig rabbit
Dinothenarus flavocephalus	[52]	1	pig
Dinothenarus fossor	[114]	1	deer
Dinothenarus pubescens	[1]	1	rat
Domene chenpengi	[3]	1	pig
Drusilla canaliculata	[1]	2	deer rat
Dysanellus sp.	[113]	1	rabbit
Edaphus sp.	[134]	1	pig
Emus hirtus	[173]	2	human pig
Eulissus chalybaeus	[57]	5	rat pig
Euplectus piceus	[1]	1	rat
Eusphalerum semicoleoptratum	[1]	1	rat
Falagria sp.	[22]	1	rat
Gabrius appendiculatus	[91]	1	pig
Gabrius microphthalamus	[68]	2	pig
Gabrius nigritulus	[54]	1	pig

Gabrius osseticus	[170]	2	pig
Gabrius trossulus	[55]	1	fish
Gabronthus mgogoricus	[155]	1	rat
Gastrisus sp.	[174]	1	rabbit
Gauropterus sp.	[175]	1	rat
Gennadota canadensis	[68]	2	pig
Geostiba circellaris	[163]	2	pig
Gnypeta sp.	[22]	1	rat
Gyrohypnus fracticornis	[13]	6	human pig deer
Gyrohypnus punctulatus	[55]	3	pig fish
Habrocerus capillaricornis	[93]	1	human
Habrocerus sp.	[127]	1	pig
Haematodes bicolor	[8]	1	pig
Haematodes sp.	[8]	1	pig
Haida sp.	[127]	1	pig
Haploglossa villosula	[164]	1	pig
Hesperus haemorrhoidalis	[83]	1	pig
Hesperus sp.	[19]	1	pig
Heterothops dissimilis	[52]	1	pig
Heterothops sp.	[69]	3	pig
Hipotelus sp.	[58]	1	pig
Homaeotarsus sellatus	[61]	1	pig
Homaeotarsus sp.	[127]	1	pig
Homalota sp.	[127]	1	pig
Hoplandria peltata	[22]	1	rat
Hoplandria sp.	[79]	2	pig rat
Hypnogyra angularis	[52]	1	pig
Ischnoglossa prolixa	[164]	1	pig
Ischnosoma sp.	[20]	1	pig deer
Lathrobium brunnipes	[163]	1	pig
Lathrobium confusum	[67]	1	pig
Lathropinus sp.	[62]	1	pig
Liogluta alpestris	[52]	3	pig
Liogluta granigera	[168]	1	pig
Lispinus sp.	[58]	1	pig
Lithocaris sp.	[95]	2	pig
Lithocharis ochracea	[37]	2	human cat
Lobrathium sp.	[105]	1	pig
Lordithon trinotatus	[114]	1	deer
Lordithon thoracicus	[13]	1	deer
Megalinus glabratus	[30]	2	pig
Megalopinus sp.	[58]	1	pig
Megarthrus angulicollis	[1]	1	rat
Megarthrus denticollis	[163]	2	pig

Megarthrus depressus	[163]	3	pig
Megarthrus nitidulus	[176]	1	pig
Megarthrus nitidulus	[163]	1	pig
Megarthrus prosseni	[163]	1	pig
Megarthrus sp.	[20]	1	pig deer
Megarthrus stercorarius	[163]	2	pig
Micropeplus staphylinoides	[43]	2	pig
Mocyta fungi	[68]	7	Pig rat
Mycetoporus longulus	[43]	2	pig
Mycetoporus lucidulus	[155]	1	rat
Mycetoporus mulsanti	[43]	2	pig
Mycetoporus nigricollis	[43]	2	pig
Mycetoporus piceolus	[43]	2	pig
Mycetoporus solidicornis	[43]	2	pig
Myllaena sp.	[22]	1	rat
Nazeris ibericus	[43]	2	pig
Nehemitropia lividipennis	[68]	3	pig rat
Neobisnius sp.	[79]	1	pig
Neohypnus obscurus	[68]	2	pig
Neohypnus sp.	[155]	3	pig rat
Neosilusa ceylonica	[19]	1	pig
Ocalea picata	[163]	2	pig
Ocyota sp.	[22]	1	rat
Ocypus aethiops	[43]	2	pig
Ocypus brunnipes	[55]	3	pig fish
Ocypus mus	[54]	2	pig
Ocypus olens	[164]	5	pig
Ocypus ophthalmicus	[170]	2	pig
Ocypus picipennis	[91]	1	pig
Ocypus sericeicollis	[52]	1	pig
Ocypus sp.	[88]	3	pig
Ocypus tenebricosus	[163]	2	pig
Oligota parva	[93]	1	human
Oligota pusillima	[43]	2	pig
Oligotergus sp.	[79]	1	pig
Olophrum assimile	[11]	2	pig
Olophrum piceum	[164]	1	pig
Omalium caesum	[1]	2	pig rat
Omalium rivulare	[23]	13	human pig rabbit rat wolf
Omalium rugatum	[163]	3	pig
Omalium septentrionis	[163]	3	pig
Omalium sp.	[155]	2	pig rat
Ontholestes cingulatus	[35]	10	human pig
Ontholestes gracilis	[16]	1	rat

Ontholestes murinus	[1]	15	human pig deer rabbit fish
Ontholestes sp.	[134]	1	pig
Ontholestes tessellatus	[1]	9	pig fish rat
Orus sp.	[20]	1	pig bear
Osorius sp.	[79]	1	pig
Othius subuliformis	[70]	3	pig
Oxypoda acuminata	[70]	2	pig
Oxypoda alternans	[1]	2	pig rat
Oxypoda brevicornis	[163]	3	pig
Oxypoda collaris	[52]	1	pig
Oxypoda cristata	[52]	1	pig
Oxypoda formosa	[114]	1	deer
Oxypoda longipes	[52]	1	pig
Oxypoda opaca	[70]	3	pig
Oxypoda vittata	[70]	1	pig
Oxytelus convergens	[4]	2	pig bear deer alligator
Oxytelus incisus	[37]	1	cat
Oxytelus laqueatus	[164]	3	pig
Oxytelus pennsylvanicus	[4]	2	deer
Oxytelus sp.	[69]	3	pig human
Paederidus ruficollis	[54]	1	pig
Paederomimus sp.	[167]	1	pig
Paederus littoralis	[11]	2	pig rabbit
Paederus sp.	[177]	3	pig
Palporus nitidulus	[43]	2	pig
Parabolitobius inclinans	[164]	1	pig
Parocyusa sp.	[22]	1	rat
Pella cognata	[163]	2	pig
Pella humeralis	[163]	3	pig rat
Pella lugens	[163]	3	pig rat
Phanerota sp.	[127]	1	pig
Phanolinus sp.	[77]	1	pig
Philhygra sp.	[68]	1	pig
Philonthus addendus	[1]	9	pig rat fish
Philonthus albipes	[164]	2	pig
Philonthus atratus	[23]	2	pig rabbit
Philonthus bonaerensis	[178]	1	pig
Philonthus caeruleipennis	[68]	3	pig
Philonthus caerulescens	[164]	1	pig
Philonthus carbonarius	[55]	7	human pig fish
Philonthus chalceus	[55]	1	fish
Philonthus cognatus	[164]	5	pig rabbit
Philonthus concinnus	[70]	6	pig
Philonthus confinis	[55]	1	fish

Philonthus corruscus	[55]	5	pig fish
Philonthus corvinus	[164]	1	pig
Philonthus crotchi	[35]	1	pig
Philonthus cruentatus	[35]	6	pig rabbit
Philonthus cyanipennis	[5]	2	human pig
Philonthus debilis	[170]	1	pig
Philonthus decorus	[164]	6	human pig
Philonthus discoideus	[37]	6	human pig cat
Philonthus ebeninus	[52]	1	pig
Philonthus figulus	[28]	1	pig
Philonthus flavolimbatus	[69]	3	pig
Philonthus furvus	[35]	1	pig
Philonthus hepaticus	[95]	4	human pig
Philonthus intermedius	[164]	3	pig deer
Philonthus japonicus	[33]	3	pig
Philonthus laevicollis	[55]	3	pig fish
Philonthus laminatus	[25]	8	pig rabbit
Philonthus lepidus	[170]	2	pig
Philonthus longicornis	[37]	14	pig rabbit cat
Philonthus marginatus	[23]	9	human pig rabbit fish
Philonthus nitidus	[163]	1	pig
Philonthus parvicornis	[70]	1	pig
Philonthus politus	[49]	20	pig gull rat rabbit deer fish
Philonthus pseudovarians	[70]	2	pig
Philonthus quisquiliarius	[164]	1	pig
Philonthus rectangulus	[37]	13	pig cat fish
Philonthus rubromaculatus	[178]	1	pig
Philonthus rufipes	[114]	1	deer
Philonthus sanguinolentus	[23]	2	pig rabbit
Philonthus sericans	[86]	5	pig
Philonthus sericinus	[68]	2	pig
Philonthus sp.	[37]	34	human pig cat rabbit rat wolf
Philonthus speciosus	[52]	1	pig
Philonthus spinipes	[70]	6	human pig
Philonthus splendens	[70]	10	human pig deer
Philonthus succicola	[1]	11	human pig deer rat
Philonthus tenuicornis	[70]	7	human pig
Philonthus umbratilis	[70]	1	pig
Philonthus umbrinus	[4]	2	pig bear deer alligator
Philonthus varians	[1]	11	pig rat deer fish
Phloenomus sp.	[22]	1	rat
Phucobius simulator	[3]	1	pig
Placusa tachyporoides	[163]	2	pig
Plataraea brunnea	[163]	1	pig

Platydracus brevicornis	[3]	1	pig
Platydracus chalcocephalus	[164]	1	pig
Platydracus cinnamopterus	[111]	2	pig turtle
Platydracus exulans	[20]	1	pig
Platydracus flavopunctatus	[54]	2	pig
Platydracus fossator	[144]	1	pig
Platydracus hypocrita	[54]	2	pig
Platydracus immaculatus	[159]	1	pig
Platydracus maculosus	[111]	7	pig bear deer alligator turtle rat
Platydracus ochropygus	[79]	2	pig
Platydracus paganus	[17]	1	pig
Platydracus stercorarius	[170]	2	pig
Platydracus sp.	[75]	8	pig rat bovine
Platystethus americanus	[67]	2	pig
Platystethus arenarius	[125]	4	Human pig deer
Platystethus capito	[11]	1	pig
Platystethus nitens	[52]	1	pig
Platystethus sp.	[2]	2	Pig wolf
Plochionocerus fulgens	[49]	2	pig gull
Plociopterus sp.	[79]	1	pig
Proteinus atomarius	[43]	2	pig
Proteinus brachypteru	[163]	3	pig
Proteinus crenulatus	[1]	1	rat
Proteinus ovalis	[11]	2	pig human
Pseudopsis sp.	[58]	1	pig
Quedius capucinus	[4]	2	pig bear deer alligator
Quedius cinctus	[68]	8	pig
Quedius dilatatus	[32]	1	pig
Quedius erythrogaster	[68]	2	pig
Quedius fuliginosus	[55]	3	pig fish
Quedius fumatus	[43]	3	pig
Quedius labradorensis	[6]	1	pig
Quedius lateralis	[164]	5	human pig
Quedius latinus	[43]	2	pig
Quedius levicollis	[13]	3	pig deer
Quedius lucidulus	[163]	2	pig
Quedius mesomelinus	[1]	7	human pig deer rat
Quedius molochinus	[55]	2	pig fish
Quedius pectinatus	[60]	1	deer
Quedius riparius	[164]	1	pig
Quedius rusticus	[68]	2	pig
Quedius scitus	[170]	1	pig
Quedius semiaenus	[164]	1	pig
Quedius sp.	[20]	6	human pig rat

Quedius vexans	[170]	1	pig
Quedius xanthopus	[170]	1	pig
Rugilus erichsonii	[55]	1	fish
Rugilus mixtus	[163]	2	pig
Rugilus orbiculatus	[1]	2	human rat
Rugilus rufipes	[55]	6	pig deer fish
Rugulus sp.	[4]	2	pig bear deer alligator
Sepedophilus sp.	[58]	3	pig deer rabbit
Silusa rubiginosa	[164]	1	pig
Staphylinini sp.	[38]	1	pig
Staphylinus erythropterus	[170]	3	pig
Stenus bifoveolatus	[43]	2	pig
Stenus bimaculatus	[55]	1	fish
Stenus clavicornis	[1]	2	pig rat
Stenus lustrator	[55]	1	fish
Stenus similis	[1]	1	rat
Stenus sp.	[62]	4	pig wolf
Stilocharis sp.	[79]	1	pig
Styngetus sp.	[167]	1	pig
Tachinus axillaris	[155]	3	pig bear deer alligator rat
Tachinus basalis	[35]	1	pig
Tachinus bipustulatus	[164]	1	pig
Tachinus corticinus	[164]	2	pig
Tachinus fumipennis	[68]	2	pig
Tachinus humeralis	[70]	3	human pig
Tachinus laticollis	[1]	9	pig deer rat
Tachinus luridus	[68]	2	pig
Tachinus marginellus	[91]	1	pig
Tachinus memnonius	[68]	2	pig
Tachinus pallipes	[13]	5	pig deer
Tachinus proximus	[164]	2	pig
Tachinus quebecensis	[68]	2	pig
Tachinus rufipes	[25]	6	pig rabbit rat
Tachinus subterraneus	[164]	1	pig
Tachyporus dispar	[68]	2	pig
Tachyporus hypnorum	[11]	1	pig
Tachyporus mexicanus	[68]	2	pig
Tachyporus pusillus	[114]	1	deer
Tasgius melanarius	[1]	2	Rat fish
Tasgius morsitans	[170]	1	pig
Thamiaraea brittoni	[4]	2	pig bear deer alligator
Thyreocephalus albertisi	[37]	3	pig cat
Vellica sp.	[127]	1	pig
Xantholinus gallicus	[164]	1	pig

		Xantholinus laevigatus	[163]	3	pig fish
		Xantholinus linearis	[55]	5	pig fish
		Xantholinus longiventris	[164]	2	pig
		Xantholinus sp.	[95]	2	pig
		Xantholinus translucidus	[43]	2	pig
		Xantholinus tricolor	[114]	1	deer
		Xanthopygus bicolor	[79]	2	pig rabbit
		Xanthopygus sp.	[57]	4	pig rabbit
		Xenopygus analis	[28]	1	pig
		Xenopygus sp.	[77]	1	pig
		unidentified	[41]	48	human pig rat rabbit bear deer alligator chicken dog bovine
Tenebrionidae	Omnivorous	*Achanius antofagastensis*	[179]	1	pig
		Alphitobius diaperinus	[70]	2	pig
		Alphitobius laevigatus	[180]	1	pig
		Blaps davidea	[40]	1	pig
		Blaps sulcata	[102]	2	rabbit
		Blapstinus punctulatus	[82]	2	pig
		Blapstinus histricus	[181]	1	pig
		Ceropria induta	[60]	1	deer
		Conibius franzi	[180]	1	pig
		Coniontis ovalis	[38]	1	pig
		Corticeus unicolor	[114]	1	deer
		Cossyphus hoffmannseggii	[43]	2	pig
		Crypticus gibbulus	[30]	2	pig
		Dailognatha quadricollis	[182]	1	rabbit
		Elodes sp.	[56]	1	rat
		Emmallodera crenaticostata	[15]	1	pig
		Emmallodera perlifera	[180]	1	pig
		Epitragus sp.	[15]	2	pig
		Eutelocera cadaverina	[180]	1	pig
		Gondwanocripticus platensis	[8]	1	pig
		Gonocephalum demestiodes	[88]	1	pig
		Gonocephalum granulatum	[30]	2	pig
		Gonocephalum pusillum	[21]	1	rabbit
		Gonocephalum sieratum	[36]	1	pig
		Gonocephalum sp.	[37]	6	pig cat rabbit chicken
		Helea castor	[147]	2	pig
		Hemasodes minutus	[180]	1	pig
		Himatismus sp.	[108]	1	rabbit
		Hylithus tentyroides	[82]	4	pig cattle horse
		Hymenorus sp.	[183]	1	rabbit
		Lagria villosa	[97]	2	pig
		Leptynoderes nordenskioldi	[180]	1	pig

		Leptynoderes strangulata	[8]	2	pig
		Lobopoda breyeri	[15]	1	pig
		Megelenophorus americanus	[82]	1	pig
		Mesostena puncticollis	[120]	1	rabbit
		Microdera kraatzi	[24]	1	rabbit
		Nyctelia circumundata	[15]	1	pig
		Ocnera andresi	[94]	1	pig
		Omopheres difficilis	[180]	1	pig
		Omopheres scabripennis	[180]	1	pig
		Patagonogeniux collaris	[15]	1	pig
		Pimelia boyeri	[24]	2	rabbit
		Pimelia interjecta	[184]	1	human
		Poecilocrypticus formicophilus	[180]	1	pig
		Praocis ecostata	[180]	1	pig
		Prionotheca coronata	[185]	1	human
		Salax lacordairei	[82]	3	pig cattle horse
		Scleron reitteri	[39]	1	rabbit
		Scotobius andrassyi	[180]	1	chicken
		Scotobius casicus	[180]	1	pig
		Scotobius clathratus	[8]	1	pig
		Scotobius granosus	[180]	1	cow
		Scotobius miliaris	[8]	2	pig
		Scotobius muricatus	[8]	1	pig
		Scotobius ovalis	[180]	1	pig
		Scotobius pilularius	[180]	2	pig
		Scotobius punctatus	[180]	1	chicken
		Scotoderus cancellatus	[182]	1	rabbit
		Stenopsis sp.	[45]	1	chicken
		Tenebrio molitor	[60]	3	human pig deer
		Tenebrio obscurus	[46]	1	human
		Tentyria sp.	[45]	1	chicken
		Thriptera kraatzi	[186]	1	rat
		Tribolium sp.	[28]	1	pig
		Trichoton roigi	[82]	3	pig
		Uloma latimanus	[59]	1	pig
		Vaniosus profana	[179]	2	pig cattle horse
		unidentified	[41]	18	human pig rat chicken dog
Trogidae	Necrophagous	*Afromorgus chinensis*	[187]	1	rabbit
		Glyptotrox spinulosus	[138]	1	pig
		Omorgus bachorum	[188]	1	pig
		Omorgus batesi	[8]	1	pig
		Omorgus candezei	[189]	1	goat
		Omorgus candidus	[103]	1	rabbit
		Omorgus ciliatus	[189]	2	pig horse goat

Omorgus loxus	[189]	1	chicken
Omorgus pastillarius	[189]	1	horse goat
Omorgus persuberosus	[131]	1	pig
Omorgus punctatus	[20]	1	pig deer
Omorgus quadrinodosus	[171]	2	human pig
Omorgus rubricans	[138]	2	pig
Omorgus suberosus	[189]	16	human pig goat cattle horse rat beer alligator cat
Omorgus tatei	[147]	3	pig rat
Omorgus sp.	[115]	8	pig bovine horse
Phoberus fascicularis	[149]	1	human
Polipochila sp.	[190]	1	pig
Polynoncus brasiliensis	[12]	1	pig
Polynoncus burmeisteri	[189]	1	goat
Polynoncus gemmingeri	[8]	1	pig
Polynoncus pedestris	[189]	1	chicken
Polynoncus sp.	[97]	2	pig rabbit
Polynoncus gemmifer	[189]	1	goat
Polynoncus guttifer	[189]	1	goat
Trox sabulosus	[1]	5	pig rat
Trox scaber	[90]	5	pig deer
Trox sp.	[92]	8	pig bovine rabbit
Trox unistriatus	[5]	3	pig
Trox variolatus	[49]	3	pig gull
unidentified	[116]	6	human pig rat

References

1. Kocarek, P. Decomposition and coleoptera succession on exposed carrion of small mammal in Opava, the Czech Republic. *Eur. J. Soil Biol.* **2003**, *39*, 31-45.
2. Bonacci, T.; Mendicino, F.; Bonelli, D.; Carlomagno, F.; Curia, G.; Scapoli, C.; Pezzi, M. Investigations on arthropods associated with decay stages of buried animals in Italy. *Insects* **2021**, *12*.
3. Park, S.; Lee, J.; Woo, D.; Ji, B.; Moon, T. Insect diversity and succession patterns on pig cadavers in Changwon, South Korea. *Entomol. Res.* **2022**, *52*, 241-250.
4. Watson E. J. and Carlton C. E. Spring succession of necrophilous insects on wildlife carcasses in Louisiana. *J. Med. Entomol.* **2003**, *40*, 338-347.
5. Gill, G.J. Decomposition and arthropod succession on above ground pig carrion in rural Manitoba. Master's Thesis, University of Manitoba, Manitoba, Canada, 2005.
6. Sampson, A.; Sikes, D.S. A preliminary forensic entomological study of beetles (Coleoptera) in interior Alaska, USA. *J. Forensic Sci.* **2020**, *65*, 2030-2035.
7. Roeglin, A.; Szentiks, C.A.; Dressler, J.; Ondruschka, B.; Schwarz, M. Entomological identification of the post-mortem colonization of wolf cadavers in different decomposition stages. *Sci. Justice* **2022**, *62*, 520-529.
8. Zanetti, N.I.; Visciarelli, E.C.; Centeno, N.D. Trophic roles of scavenger beetles in relation to decomposition stages and seasons. *Rev. Bras. Entomol.* **2015**, *59*, 132-137.
9. Mayer, A.C.G.; Vasconcelos, S.D. Necrophagous beetles associated with carcasses in a semi-arid environment

in Northeastern Brazil: Implications for forensic entomology. *Forensic Sci. Int.* **2013**, *226*, 41-45.

10. Bonacci, T.; Brandmayr, T.Z.; Brandmayr, P.; Vercillo, V.; Porcelli, F. Successional patterns of the insect fauna on a pig carcass in southern Italy and the role of *Crematogaster scutellaris* (Hymenoptera, Formicidae) as a carrion invader. *Entomol. Sci.* **2011**, *14*, 125-132.
11. Anton, E.; Niederegger, S.; Beutel, R.G. Beetles and flies collected on pig carrion in an experimental setting in Thuringia and their forensic implications. *Med. Vet. Entomol.* **2011**, *25*, 353-364.
12. Ries, A.C.R.; Costa-Silva, V.; Dos Santos, C.F.; Blochtein, B.; Thyssen, P.J. Factors affecting the composition and succession of beetles in exposed pig carcasses in southern Brazil. *J. Med. Entomol.* **2021**, *58*, 104-113.
13. Melis, C.; Teurlings, I.; Linnell, J.; Andersen, R.; Bordoni, A. Influence of a deer carcass on Coleopteran diversity in a Scandinavian boreal forest: a preliminary study. *Eur. J. Wildlife Res.* **2004**, *50*, 146-149.
14. Salimi, M.; Chatrabgoun, O.; Akbarzadeh, K.; Oshaghi, M.; Falahati, M.H.; Rafizadeh, S.; Yusuf, M.A.; Rassi, Y. Evaluation of insect succession patterns and carcass weight loss for the estimation of postmortem interval. *J. Med. Entomol.* **2018**, *55*, 1410-1422.
15. Armani A.P., Centeno N.D. and Dahinten S.L. First study of cadaveric arthropod fauna on porcine experimental models in northeastern Chubut province, Argentina. *Rev. Soc. Entomol. Argent.* **2015**, *74*, 123-132.
16. ITO, M. Study of community assembly patterns and interspecific interactions involved in insect succession on rat carcasses. *Entomol. Sci.* **2020**, *23*, 105-116.
17. Zheng, Z.; Yin, M. A forensic entomological study using pig carrions in different seasons and the exposed extent. *Korean Police Studies Review* **2013**, *12*, 335-354.
18. De Jong, G.D.; Chadwick, J.W. Decomposition and arthropod succession on exposed rabbit carrion during summer at high altitudes in Colorado, USA. *J. Med. Entomol.* **1999**, *36*, 833-845.
19. Lyu Z., Wan L., Yang Y., Tang R.; Xu L. A checklist of beetles (Insecta, Coleoptera) on pig carcasses in the suburban area of southwestern China: A preliminary study and its forensic relevance. *J. Forensic Leg. Med.* **2016**, *41*, 42-48.
20. Watson E.J.; Carlton C.E. Insect succession and decomposition of wildlife carcasses during fall and winter in Louisiana. *J. Med. Entomol.* **2005**, *42*, 193-203.
21. Yang L., Cai J., Lan L., Jiang Y., Li X.; Li J. et al. Succession of sarcosaphagous insects at summer and autumn in Shijiazhuang area. *Fa yi xue za zhi*. **2010**, *26*, 253-256.
22. Caballero U.; Leon-Cortes J. L. High diversity beetle assemblages attracted to carrion and dung in threatened tropical oak forests in Southern Mexico. *J. Insect Conserv.* **2012**, *16*, 537-547.
23. Bourel B., Martin-Bouyer L., Hedouin V., Cailliez J. C., Derout D.; Gosset D. Necrophilous insect succession on rabbit carrion in sand dune habitats in northern France. *J. Med. Entomol.* **1999**, *36*, 420-425.
24. Al-Khalifa M., Mashaly A.; Al-Qahtni A. Impacts of antemortem ingestion of alcoholic beverages on insect successional patterns. *Saudi J. Biol. Sci.* **2021**, *28*, 685-692.
25. Chapman R.F.; Sankey J. The larger invertebrate fauna of 3 rabbit carcasses. *J. Anim. Ecol.* **1955**, *24*, 395-402.
26. Zou T., Feng D., Huang G., Sun D.; Dai S. Species composition and succession of necrophagous insects on small buried baits in China. *J. Med. Entomol.* **2022**, *59*, 1182-1190.
27. Honda J.Y., Brundage A., Happy C., Kelly S.C.; Melinek J. New records of carrion feeding insects collected on human remains. *Pan-Pacific Entomol.***2008**, *84*, 29-32.
28. Santos W.E., Alves A. C.F.; Creao-Duarte A.J. Beetles (Insecta, Coleoptera) associated with pig carcasses exposed in a Caatinga area, Northeastern Brazil. *Braz. J. Biol.* **2014**, *74*, 649-655.
29. Castro M., Centeno N.; Gonzalez-Vainer P. An initial study of insect succession on pig carcasses in open pastures in the northwest of Uruguay. *Forensic Sci. Int.* **2019**, *302*.

30. Diaz-Aranda L.M., Martin-Vega D., Gomez-Gomez A., Cifrian B.; Baz A. Annual variation in decomposition and insect succession at a periurban area of central Iberian Peninsula. *J. Forensic Leg Med*. **2018**, *56*, 21-31.
31. Vanin S., Zanotti E., Gibelli D., Taborelli A., Andreola S.; Cattaneo C. Decomposition and entomological colonization of charred bodies - a pilot study. *Croat. Med. J.* **2013**, *54*, 387-393.
32. Zheng, Z.; Yin, M. Arthropod succession and decomposition patterns of pig carrions varying with the exposed extent of the carrions. *Journal of Life Science* **2011**, *21*, 1168-1175.
33. Zheng, Z.; Yin, M. A Study on the arthropod succession in exposed pig carrion. *Journal of Life Science* **2008**, *18*, 1400-1409.
34. Dekeirsschieter J., Frederickx C., Verheggen F. J., Boxho P.; Haubruge E. Forensic entomology investigations from doctor Marcel Leclercq (1924-2008): a review of cases from 1969 to 2005. *J. Med. Entomol.* **2013**, *50*, 935-954.
35. VanLaerhoven, S.L. Successional biodiversity in insect species on buried carrion in the Vancouver and Cariboo regions of British Columbia. Master's Thesis, Simon Fraser University, British Columbia, Canada, 1997.
36. Richards E.N.; Goff M.L. Arthropod succession on exposed carrion in three contrasting tropical habitats on Hawaii island, Hawaii. *J. Med. Entomol.* **1997**, *34*, 328-339.
37. Early, M. Arthropod succession patterns in exposed carrion in Hawaii. Master's Thesis, University of Hawai'i at Manoa, HI, USA, 1984.
38. Dillon, L.C. Insect succession on carrion in three biogeoclimatic zones of British Columbia. Master's Thesis, Simon Fraser University, British Columbia, Canada, 1997.
39. Bharti, M.; Singh, D. Insect faunal succession on decaying rabbit carcasses in Punjab, India. *J. Forensic Sci.* **2003**, *48*, 1133-1143.
40. Hu, G.; Kang, C.; Zhu, R.; Guo, Y.; Li, L.; Wang, Y.; Zhang, Y.; Wang, Y.; Wang, J. A preliminary study of body decomposition and arthropod succession in an arid area in northwest China during summer. *J. Med. Entomol.* **2023**, *60*, 306-315.
41. Davis, J.B. Decomposition Patterns in Terrestrial and Intertidal Habitats on O'ahu Island and Coconut Island (Moku O Loe), Hawai'i. Master's Thesis, University of Hawai'i at Manoa, Honolulu, HI, USA, 1998.
42. Bourel, B.; Hubert, N.; Hedouin, V.; Gosset, D. Forensic entomology applied to a mummified corpse. *Ann. Soc. Entomol. Fr.* **2000**, *36*, 287-290.
43. Castro, C.B.D.P. Seasonal carrion Diptera and Coleoptera communities from Lisbon (Portugal) and the utility of forensic entomology in legal medicine. Master's Thesis, Universidade de Lisboa , Portugal, 2011.
44. Kadej, M.; Szleszkowski, L.; Thannhauser, A.; Jurek, T. A mummified human corpse and associated insects of forensic importance in indoor conditions. *Int. J. Legal Med.* **2020**, *134*, 1963-1971.
45. Arnaldos, M.I.; Romera, E.; Presa, J.J.; Luna, A.; Garcia, M.D. Studies on seasonal arthropod succession on carrion in the southeastern Iberian Peninsula. *Int. J. Legal Med.* **2004**, *118*, 197-205.
46. Easton, A.M.; Smith, K.G. The entomology of the cadaver. *Medicine, science, and the law* **1970**, *10*, 208-15.
47. Perez-Marcos, M.; Isabel Arnaldos-Sanabria, M.; Dolores Garcia, M.; Jose Presa, J. Examining the sarcosaprophagous fauna in a natural mountain environment (Sierra Espuna, Murcia, Spain). *Ann. Soc. Entomol. Fr.* **2016**, *52*, 264-280.
48. Charabidze, D.; Colard, T.; Vincent, B.; Pasquerault, T.; Hedouin, V. Involvement of larder beetles (Coleoptera: Dermestidae) on human cadavers: a review of 81 forensic cases. *Int. J. Legal Med.* **2014**, *128*, 1021-1030.
49. Lord, W.D. The ecology of carrion communities in maritime-terrestrial habitats: An insular study of gull, seal, and rodent decomposition on the isles of shoals (new hampshire). Master's Thesis, University of New Hampshire, New Hampshire, USA, 1982.

50. Jiron, L.F.; Cartin, V.M. Insect succession in the decomposition of a mammal in Costa-Rica. *Journal of the New York Entomological Society* **1981**, *89*, 158-165.
51. Park, J.; Kim, C.; Do, Y. Post mortem insect colonization and body weight loss in rabbit carcasses. *Entomol. Res.* **2020**, *50*, 594-600.
52. Altunsoy, F.; Turan, Y.; Firat, S.; Sert, O. Differences in succession of Coleoptera species attracted to pig carcasses in rural and urban habitats in Eskisehir Province, Turkey. *Turk. Entomol. Derg.-Tu.* **2017**, *41*, 177-195.
53. Martin-Vega, D.; Baz, A. Spatiotemporal distribution of *Necrophagous* beetles (Coleoptera: Dermestidae, Silphidae) assemblages in natural habitats of central Spain. *Ann. Entomol. Soc. Am.* **2012**, *105*, 44-53.
54. Oezdemir, S.; Sert, O. Determination of Coleoptera fauna on carcasses in Ankara province, Turkey. *Forensic Sci. Int.* **2009**, *183*, 24-32.
55. Ulrich, W.; Zalewski, M.; Komosinski, K. Diversity of carrion visiting beetles at rural and urban sites. *Community Ecol.* **2007**, *8*, 171-181.
56. De Jong, G.D.; Hoback, W.W. Effect of investigator disturbance in experimental forensic entomology: succession and community composition. *Med. Vet. Entomol.* **2006**, *20*, 248-258.
57. DeSouza, A.M.; Linhares, A.X. Diptera and Coleoptera of potential forensic importance in southeastern Brazil: Relative abundance and seasonality. *Med. Vet. Entomol.* **1997**, *11*, 8-12.
58. Wolff M., Uribe A., Ortiz A.; Duque P. A preliminary study of forensic entomology in Medellin, Colombia. *Forensic Sci.Int.* **2001**, *120*, 53-59.
59. Park W. B., Park J. K.; Do Y. Refining decomposition stage estimation: A study of seasonal insect composition and carcass weight change. *Entomol. Res.* **2001**, *53*, 414-425.
60. Yoon J. H., Jung C. M.; Park J. Diversity on necrophagous insect of the water deer carcass decaying. *Korean Journal of Applied Entomology* **2022**, *61*, 239-248.
61. Hobischak, N.R. Freshwater invertebrate succession and decompositional studies on carrion in British Columbia. Master's Thesis, Simon Fraser University, British Columbia, Canada, 1997.
62. Martinez, E.; Duque, P.; Wolff, M. Succession pattern of carrion-feeding insects in Paramo, Colombia. *Forensic Sci. Int.* **2007**, *166*, 182-189.
63. Dalal, J.; Sharma, S.; Bhardwaj, T.; Dhattarwal, S.K.; Verma, K. A seasonal study of the decomposition pattern and insects on submerged rabbit carcases. *Orient. Insects* **2021**, *55*, 280-292.
64. Oses-Rivera, C.A.; Astesiano, E.C.T. First report of *Rhantus validus* Sharp (Coleoptera: Dytiscidae) as necrophage and generator of postmortem artifacts in a human corpse found in an artificial freshwater pond from the Región de La Araucanía, Chile. *Revista chilena de entomología* **2020**, *46*, 81-86.
65. Barrios, M.; Wolff, M. Initial study of arthropods succession and pig carrion decomposition in two freshwater ecosystems in the Colombian Andes. *Forensic Sci. Int.* **2011**, *212*, 164-172.
66. Vance G. M., Vandyk J. K.; Rowley W. A. Device for sampling aquatic insects associated with carrion in water. *J. Forensic Sci.* **1995**, *40*, 479-482.
67. Michaud, J.; Majka, C.G.; Prive, J.; Moreau, G. Natural and anthropogenic changes in the insect fauna associated with carcasses in the North American Maritime lowlands. *Forensic Sci. Int.* **2010**, *202*, 64-70.
68. Michaud, J. La vie après la mort: Étude de la succession hétérotrophe sur les carcasses de porcs domestiques dans le sud-est du Nouveau-Brunswick. Master's Thesis, Universite de Moncton, New Brunswick, Canada, 2009.
69. Rosa, T.A.; Babata, M.L.Y.; de Souza, C.M.; de Sousa, D.; de Mello-Patiu, C.A.; Vaz-de-Mello, F.Z.; Mendes, J. Arthropods associated with pig carrion in two vegetation profiles of Cerrado in the State of Minas Gerais, Brazil. *Rev. Bras. Entomol.* **2011**, *55*, 424-434.

70. Matuszewski, S.; Bajerlein, D.; Konwerski, S.; Szpila, K. An initial study of insect succession and carrion decomposition in various forest habitats of Central Europe. *Forensic Sci. Int.* **2008**, *180*, 61-69.
71. Saloña-Bordas, M.I.; de la Puebla, P.B.; Martín, B.D.; Sumner, J.; Perotti, M.A. *Ixodes ricinus* (Ixodidae), an occasional phoront on necrophagous and coprophagous beetles in Europe. *Exp. Appl. Acarol.* **2015**, *65*, 243-248.
72. Bajerlein, D.; Matuszewski, S.; Konwerski, S. Insect succession on carrion: Seasonality, habitat preference and residency of histerid beetles (Coleoptera: Histeridae) visiting pig carrion exposed in various forests (western Poland). *Pol. J. Ecol.* **2011**, *59*, 787-797.
73. Leivas, F.W.T.; Mise, K.M.; Almeida, L.M.; Macari, B.P.; Gomy, Y. New species and key of Aeletes Horn (Coleoptera: Histeridae: Abraeinae) from Brazil. *Zootaxa* **2012**, 63-68.
74. Mise, K.M.; Correa, R.C.; Almeida, L.M. Coleopterofauna found on fresh and frozen rabbit carcasses in Curitiba, Parana, Brazil. *Braz. J. Biol.* **2013**, *73*, 543-548.
75. Wang, J.; Li, Z.; Chen, Y.; Chen, Q.; Yin, X. The succession and development of insects on pig carcasses and their significances in estimating PMI in south China. *Forensic Sci. Int.* **2008**, *179*, 11-18.
76. Hildebrand, C.S. A Checklist of Forensically Important Insect Taxa on Decomposing Carcasses and Successional Patterns Related to Decomposition and Season in Minnesota. Master's Thesis, University of Minnesota, Minnesota, USA, 2019.
77. Caballero, U.; Leon-Cortes, J.L. Beetle succession and diversity between clothed sun-exposed and shaded pig carrion in a tropical dry forest landscape in Southern Mexico. *Forensic Sci. Int.* **2014**, *245*, 143-150.
78. Aballay, F.H.; Arriagada, G.; Flores, G.E.; Centeno, N.D. An illustrated key to and diagnoses of the species of Histeridae (Coleoptera) associated with decaying carcasses in Argentina. *Zookeys* **2013**, 61-84.
79. Miseo, K.M.; Barros De Souza, A.S.; Campos, C.D.M.; Ferreira Keppler, R.L.; de Almeida, L.M. Coleoptera associated with pig carcass exposed in a forest reserve, Manaus, Amazonas, Brazil. *Biota Neotrop.* **2010**, *10*, 321-324.
80. Ortloff, A.; Pena, P.; Riquelme, M. Preliminary study of the succession pattern of necrobiont insects, colonising species and larvae on pig carcasses in Temuco (Chile) for forensic applications. *Forensic Sci. Int.* **2012**, *222*, E36-E41.
81. Aballay, F.H.; Jofré, F.N.; Centeno, N.D. Asociación y estratificación de la entomofauna cadavérica a diferentes profundidades en el suelo como indicadores complementarios en largos intervalos post mortem. *Revista del Museo Argentino de Ciencias Naturales* **2017**, *19*, 225-234.
82. Aballay, F.H.; Murúa, A.F.; Acosta, J.C.; Centeno, N. First record of cadaverous arthropod fauna in human and animal substrates in San Juan, Argentina. *Revista de la Sociedad Entomológica Argentina* **2008**, *67*, 157-163.
83. Archer, M. Comparative analysis of insect succession data from Victoria (Australia) using summary statistics versus preceding mean ambient temperature models. *J. Forensic Sci.* **2014**, *59*, 404-412.
84. Taleb, M.; Tail, G.; Djedouani, B.; Acikgoz, H.N. Impact of plastic wrapping on carcass decomposition and arthropod colonisation in northern Africa during spring. *Sci. Justice* **2022**, *62*, 117-127.
85. Celli, N.G.R.; Leivas, F.W.T.; Caneparo, M.F.C.; Almeida, L.M. Identification key and diagnosis of forensic interest Histeridae (Insecta: Coleoptera) from Brazil. *Iheringia Ser. Zool.* **2015**, *105*, 461-473.
86. Tabor, K.L.; Fell, R.D.; Brewster, C.C. Insect fauna visiting carrion in Southwest Virginia. *Forensic Sci. Int.* **2005**, *150*, 73-80.
87. Faria L. S., Paseto M. L., Couri M. S., Mello-Patiu C. A.; Mendes J. Insects Associated with Pig Carrion in Two Environments of the Brazilian Savanna. *Neotrop. Entomol.* **2018**, *47*, 181-198.
88. Ekanem M. S.; Dike M. C. Arthropod succession on pig carcasses in southeastern Nigeria. *Papéis Avulsos de Zoologia (São Paulo)*. **2010**, *50*, 561-570.

89. Vasconcelos S. D.; Araujo M. C. S. Necrophagous species of Diptera and Coleoptera in northeastern Brazil: state of the art and challenges for the Forensic Entomologist. *Rev. Bras. Entomol.* **2012**, *56*, 7-14.
90. Matuszewski S., Bajerlein D., Konwerski S.; Szpila K. Insect succession and carrion decomposition in selected forests of Central Europe. Part 2: Composition and residency patterns of carrion fauna. *Forensic Sci. Int.* **2010**, *195*, 42-51.
91. Madra A., Fratczak K., Grzywacz A.; Matuszewski S. Long-term study of pig carrion entomofauna. *Forensic Sci. Int.* **2015**, *252*, 1-10.
92. Tabor K. L., Brewster C. C.; Fell R. D. Analysis of the successional patterns of insects on carrion in southwest Virginia. *J. Med. Entomol.* **2004**, *41*, 785-795.
93. Salona M. I., Lourdes Moraza M., Carles-Tolra M., Iraola V., Bahillo P.; Yelamos T. et al. Searching the Soil: Forensic importance of edaphic fauna after the removal of a corpse. *J. Forensic Sci.* **2010**, *55*, 1652-1655.
94. Abd El-Bar M. M.; Sawaby R. F. A preliminary investigation of insect colonization and succession on remains of rabbits treated with an organophosphate insecticide in El-Qalyubiya Governorate of Egypt. *Forensic Sci.Int.* **2011**, *208*, E26-E30.
95. Guarin Vargas, E.G. Insectos de importancia forense asociados a la descomposición cadavérica del cerdo Sus domesticus, expuesto a sol, sombra total y sombra parcial, en Mayagüez, Puerto Rico. Master's Thesis, University of Puerto Rico, Mayaguez, USA, 2006.
96. Correa R. C., Moura D. P., Leivas F. W. T.; Almeida L. M. *Operclipygus hospes* (Lewis) (Coleoptera, Histeridae): a beetle of potential forensic importance for buried bodies. *Neotrop. Entomol.* **2012**, *41*, 254-256.
97. Mise K. M., de Almelda L. M.; Moura M. O. A study of the Coleoptera (Insecta) fauna that inhabits *Sus scrofa* L carcass in Curitiba, Parana. *Rev. Bras. Entomol.* **2007**, *51*, 358-368.
98. Pastula, E.C. Insect timing and succession on buried carrion in East Lansing, Master's Thesis, Michigan. Michigan State University, Michigan, USA, 2012.
99. Introna F., Campobasso C. P.; Di Fazio A. Three case studies in forensic entomology from Southern Italy. *J. Forensic Sci.* **1998**, *43*, 210-214.
100. Aneyo I., Alafia O., Doherty F., Udoma R., Balogun B.; Adeola A. Aerobic microbe community and necrophagous insects associated with decomposition of pig carrion poisoned with lead. *Legal Med-Tokyo* **2020**, *42*.
101. Sebastiao M.; Prado E Castro C. A preliminary study of carrion insects and their succession in Luanda, Angola. *J. Med. Entomol.* **2019**, *56*, 378-383.
102. Tantawi T. I., Elkady E. M., Greenberg B.; ElGhaffar H. A. Arthropod succession on exposed rabbit carrion in Alexandria, Egypt. *J. Med. Entomol.* **1996**, *33*, 566-580.
103. Barton P. S., Evans M. J., Pechal J. L.; Benbow M. E. Necrophilous insect dynamics at small vertebrate carrion in a temperate eucalypt woodland. *J. Med. Entomol.* **2017**, *54*, 964-973.
104. Aballay F.H., Murua A.F., Acosta J.C.; Centeno N.D. Succession of carrion fauna in the arid region of San Juan province, Argentina and its forensic relevance. *Neotrop. Entomol.* **2012**, *41*, 27-31.
105. Sharanowski B.J., Walker E.G.; Anderson G. S. Insect succession and decomposition patterns on shaded and sunlit carrion in Saskatchewan in three different seasons. *Forensic Sci. Int.* **2008**, *179*, 219-240.
106. Farrell J.F., Whittington A.E.; Zalucki M.P. A review of necrophagous insects colonising human and animal cadavers in south-east Queensland, Australia. *Forensic Sci.Int.* **2015**, *257*, 149-154.
107. Shayya S., Degallier N., Nel A., Azar D.; Lackner T. Contribution to the knowledge of *Saprinus* Erichson, 1834 of forensic relevance from Lebanon (Coleoptera, Histeridae). *Zookeys* **2018**, 117-152.
108. Abouzied E.M. Insect colonization and succession on rabbit carcasses in southwestern mountains of the Kingdom of Saudi Arabia. *J. Med. Entomol.* **2014**, *51*, 1168-1174.

109. Grow K.M. The faunal succession of forensically important arthropods and large vertebrate scavengers in rural Northwest Florida. Master's Thesis, The University of West Florida, Florida, USA, 2017.
110. Su, R.N.; Guo, Y.D.; Xie, D.; Peng, Y.L.; Cai, J.F.; Hua, F.; Sheng, L.H. Identification of forensically important beetles (Coleoptera: Histeridae) in China based on 16S rRNA and Cyt b. *Trop. Biomed.* **2013**, *30*, 375-387.
111. Aballay, F.H.; Murua, A.F.; Acosta, J.C.; Centeno, N.D. Succession of carrion fauna in the arid region of San Juan Province, Argentina and its forensic relevance. *Neotrop. Entomol.* **2012**, *41*, 27-31.
112. Correa, R.C.; Caneparo, M.; Vairo, K.P.; de Lara, A.G.; Moura, M.O. What have we learned from the dead? A compilation of three years of cooperation between entomologists and crime scene investigators in Southern Brazil. *Rev. Bras. Entomol.* **2019**, *63*, 224-231.
113. Correa, R.C.; Almeida, L.M.; Moura, M.O. Coleoptera associated with buried carrion: potential forensic importance and seasonal composition. *J. Med. Entomol.* **2014**, *51*, 1057-1066.
114. von Hoermann, C.; Lackner, T.; Sommer, D.; Heurich, M.; Benbow, M.E.; Mueller, J. Carcasses at Fixed Locations Host a Higher Diversity of Necrophilous Beetles. *Insects* **2021**, *12*.
115. Archer, M.S.; Elgar, M.A. Yearly activity patterns in southern Victoria (Australia) of seasonally active carrion insects. *Forensic Sci. Int.* **2003**, *132*, 173-176.
116. Moura, M.O.; DeCarvalho, C.; Monteiro, E. A preliminary analysis of insects of medico-legal importance in Curitiba, State of Parana. *Mem. I. Oswaldo Cruz* **1997**, *92*, 269-274.
117. Hobischak, N.R.; VanLaerhoven, S.L.; Anderson, G.S. Successional patterns of diversity in insect fauna on carrion in sun and shade in the Boreal Forest Region of Canada, near Edmonton, Alberta. *Can. Entomol.* **2006**, *138*, 376-383.
118. Archer, M.S. Annual variation in arrival and departure times of carrion insects at carcasses: implications for succession studies in forensic entomology. *Aust. J. Zool.* **2003**, *51*, 569-576.
119. Oliva, A. Insects of forensic significance in Argentina. *Forensic Sci. Int.* **2001**, *120*, 145-154.
120. Defilippo, F.; Munari, M.; Grisendi, A.; Gaudio, R.M.; D'Incau, M.; Lavazza, A.; Rubini, S. Insect colonisation and the decomposition process in aerated versus watertight burial systems. *Insects* **2023**, *14*.
121. Adair, T.W.; Kondratieff, B.C. The occurrence of *Nitidula flavomaculata* (Coleoptera: Nitidulidae) on a human corpse. *Entomol. News* **1996**, *107*, 233-236.
122. De Jong, G.D.; Chadwick, J.W. New northern records for *Necrodes surinamensis* (Coleoptera : Silphidae) and *Nitidula nigra* (Coleoptera : Nitidulidae) from the Northwest Territories, Canada. *Entomol. News* **1999**, *110*, 236-236.
123. Williams, K.A.; Clitheroe, C.L.; Villet, M.H.; Midgley, J.M. The first record of *Omosita nearctica* Kirejtshuk (Coleoptera, Nitidulidae) in South Africa, with the first description of its mature larva. *Afr. Invertebr.* **2021**, *62*, 257-271.
124. Bonacci, T.; Greco, S.; Brandmayr, T.Z. Insect fauna and degradation activity of *Thanatophilus* species on carrion in southern Italy (Coleoptera: Silphidae). *Entomol. Gen.* **2011**, *33*, 63-70.
125. van Klink R., Van Laar-Wiersma J., Vorst O.; Smit C. Rewilding with large herbivores: Positive direct and delayed effects of carrion on plant and arthropod communities. *PLoS One*. **2020**, *15,* e0226946.
126. Braack L. Community dynamics of carrion-attendant arthropods in tropical african woodland. *Oecologia*. **1987**, *72*, 402-409.
127. Turner J. Insects associated with shallow-trench composting of swine carcasses from a simulated African swine fever disease outbreak and potential for virus transmission. Master's Thesis, Oklahoma State University, USA, 2021.
128. Engasser, E.L.; Stone, R.L.; Jameson, M.L. Habitat Associations of Carrion Beetles (Coleoptera: Silphidae) Across a Full Annual Cycle. *Environ. Entomol.* **2021**, *50*, 605-614.

129. Campos, R.C.; Medina Hernandez, M.I. Dung beetle assemblages (Coleoptera, Scarabaeinae) in Atlantic forest fragments in southern Brazil. *Rev. Bras. Entomol.* **2013**, *57*, 47-54.
130. Silva, F.; Hernandez, M.; Ide, S.; de Moura, R.D. Copro-necrophagous scarab community (Coleoptera, Scarabaeidae) of the Brejo Novo region, Caruaru, Pernambuco, Brazil. *Rev. Bras. Entomol.* **2007**, *51*, 228-233.
131. Eulalio, A.D.M.D.; Paula-Silva, M.C.D.; Michelutti, K.B.; de Oliveira, F.C.; Brum, A.C.D.S.; Lima-Junior, S.E.; Cardoso, C.A.L.; Antonialli-Junior, W.F. Effect of thiamethoxam (organophosphate) on the flies and beetle visitation and cadaveric decomposition process. *Rev. Bras. Entomol.* **2023**, *67*.
132. Andrade-Herrera, K.; Ruiz-González, C.; Córdova-Espinoza, M. Comparative study of insects associated with guinea pig corpses in two forms of death in Castilla, Piura (Perú). *Cuadernos de Medicina Forense* **2018**, *24*, 6-13.
133. Carvalho, L.; Thyssen, P.J.; Linhares, A.X.; Palhares, F. A checklist of arthropods associated with pig carrion and human corpses in southeastern Brazil. *Mem. I. Oswaldo Cruz* **2000**, *95*, 135-138.
134. Perez, S.P.; Duque, P.; Wolff, M. Successional behavior and occurrence matrix of carrion-associated arthropods in the urban area of Medellin, Colombia. *J. Forensic Sci.* **2005**, *50*, 448-454.
135. Ururahy-Rodrigues, A.; Rafael, J.A.; Wanderley, R.F.; Marques, H.; Pujol-Luz, J.R. *Coprophanaeus lancifer* (Linnaeus, 1767) (Coleoptera, Scarabaeidae) activity moves a man-size pig carcass: Relevant data for forensic taphonomy. *Forensic Sci. Int.* **2008**, *182*, E19-E22.
136. Gross S. D. Carrion-associated arthropods in rural and urban environments. Master's Thesis, Purdue University, Indiana, UAS, 2015.
137. Velasquez, Y.A checklist of arthropods associated with rat carrion in a montane locality of northern Venezuela. *Forensic Sci. Int.* **2008**, *174*, 67-69.
138. González-Hernández A.L., Navarrete-Heredia J.L., Quiroz-Rocha G.A.; López-Caro J.B. Beetles (Scarabaeidae, Trogidae and Silphidae) associated to a piglet carcass *Sus scrofa* (Linnaeus, 1758) from Los Colomos forest, Guadalajara, Jalisco. *Acta zoológica mexicana*. **2013**, *29*, 252-254.
139. Midgley, J.M.; Collett, I.J.; Villet, M.H. The distribution, habitat, diet and forensic significance of the scarab *Frankenbergerius forcipatus* (Harold, 1881) (Coleoptera: Scarabaeidae). *Afr. Invertebr.* **2012**, *53*, 745-749.
140. Mashaly, A.M.A. Entomofaunal succession patterns on burnt and unburnt rabbit carrion. *J. Med. Entomol.* **2016**, *53*, 296-303.
141. Kientega H.D., Ilboudo E. M.M., Waongo A., Ilboudo Z., Zeba M. T.A.; Sanon A. Seasonal diversity and dynamics of entomofauna associated with the decomposition of pig (*Sus scrofa domesticus* L.) carcasses in a Peri-urban area of Central Burkina Faso, West Africa. *Forensic Sci. Med. Pathol.* **2024**, *20*, 89-99.
142. Voss, S.C.; Cook, D.F.; Dadour, I.R. Decomposition and insect succession of clothed and unclothed carcasses in Western Australia. *Forensic Sci. Int.* **2011**, *211*, 67-75.
143. Tembe, D.; Mukaratirwa, S. Insect succession and decomposition pattern on pig carrion during warm and cold seasons in Kwazulu-Natal Province of South Africa. *J. Med. Entomol.* **2021**, *58*, 2047-2057.
144. Li, L.; Guo, Y.; Zhou, Y.; Yang, Y.; Kang, C.; Hu, G.; Wang, Y.; Zhang, Y.; Wang, Y.; Jiangfeng, W. Succession patterns of sarcosaprophagous insects on pig carcasses in different months in Yangtze River Delta, China. *Forensic Sci. Int.* **2023**, *342, 111518*.
145. Cruise, A.; Watson, D.W.; Schal, C.; Doucet, D. Ecological succession of adult necrophilous insects on neonate *Sus scrofa* domesticus in central North Carolina. *Plos One* **2018**, *13*, e0195785.
146. Ellison G. The effect of scavenger mutilation on insect succession at impala carcasses in Southern Africa. *J. Zool.* **1990**, *220*, 679-688.
147. Voss, S.C.; Forbes, S.L.; Dadour, I.R. Decomposition and insect succession on cadavers inside a vehicle environment. *Forensic Sci Med Pat.* **2008**, *4*, 22-32.

148. Alhag S. K. et al. Patterns of insect succession and decomposition on rabbit carcasses over the summer and winter seasons. *Int. J. Trop. Insect Sci.* **2023**, *43*, 1715-1722.
149. Ridgeway J.A., Midgley J.M., Collett I.J.; Villet M.H. Advantages of using development models of the carrion beetles *Thanatophilus micans* (Fabricius) and *T. mutilatus* (Castelneau) (Coleoptera: Silphidae) for estimating minimum post mortem intervals, verified with case data. *Int. J. Legal Med.* **2014**, *128*, 207-220.
150. Messas Y.F., Souza H.S., Schiffler G.; Sobezak J.F. First record of necrophagy by *Scybalocanthon nigriceps* Harold (Coleoptera, Scarabaeidae, Scarabaeinae). *Rev. Bras. Entomol.* **2012**, *56*, 257-258.
151. Luo, Y.; Meng, F. Identification of Forensically Important Carrion Beetles (Coleoptera: Staphilinidae) in China Based on COI and COII. *J. Med. Entomol.* **2023**, *60*, 24-31.
152. Thuemmel, L.; Lutz, L.; Geissenberger, J.; Pittner, S.; Heimer, J.; Amendt, J. Decomposition and insect succession of pig cadavers in tents versus outdoors-A preliminary study. *Forensic Sci. Int.* **2023**, *346*.
153. Putman R.J. Role of carrion frequenting arthropods in decay process. *Ecol. Entomol.* **1978**, *3*, 133-139.
154. Zheng, Z., Park, S., Song, T.; Xuan, Z. A Study on decomposition and insect fauna of carrions exposed to the sun and shade. *Korean Police Studies Review* **2015**, *14*, 539-556.
155. Tomberlin, J.K.; Adler, P.H. Seasonal colonization and decomposition of rat carrion in water and on land in an open field in South Carolina. *J. Med. Entomol.* **1998**, *35*, 704-9.
156. Zheng, Z.; Yin, M. Decomposition pattern and entomofauna in the pig carcasses in different conditions. *Journal of Science Criminal Investigation* **2016**, *10*, 187-194.
157. Schoenly, K.G.; Haskell, N.H.; Hall, R.D.; Gbur, J.R. Comparative performance and complementarity of four sampling methods and arthropod preference tests from human and porcine remains at the forensic anthropology center in Knoxville, Tennessee. *J. Med. Entomol.* **2007**, *44*, 881-894.
158. Rintoul, D.A.; Krueger, L.M.; Woodard, C.; Throne, J.E. Carrion beetles (Coleoptera : Silphidae) of the Konza Prairie Biological Station. *J. Kansas Entomol. Soc.* **2005**, *78*, 124-133.
159. Shahid, S.A.; Schoenly, K.; Haskell, N.H.; Hall, R.D.; Zhang, W.J. Carcass enrichment does not alter decay rates or arthropod community structure: A test of the arthropod saturation hypothesis at the Anthropology Research Facility in Knoxville, Tennessee. *J. Med. Entomol.* **2003**, *40*, 559-569.
160. Castillo P., Sanabria C.; Monroy F. Insectos de importancia forense en cadáveres de cerdo (sus scrofa) en la paz Bolivia. *Medicina Legal de Costa Rica*. **34**, 26-34 (2017).
161. Tosti-Croce E., Oses C., Ortloff A.; De La Fuente J.C. Morphological description and differentation of two instars of *Oxelytrum lineatocolle* larvae (Laporte, 1840) (Coleoptera: Silphidae). *Gayana*. **2014**, *78*, 130-134.
162. King, J.E.; Riegler, M.; Thomas, R.G.; Spooner-Hart, R.N. Phylogenetic placement of Australian carrion beetles (Coleoptera: Silphidae). *Austral Entomol* **2015**, *54*, 366-375.
163. Weithmann, S.; Kuppler, J.; Degasperi, G.; Steiger, S.; Ayasse, M.; von Hoermann, C. Local and landscape effects on carrion-associated rove beetle (Coleoptera: Staphylinidae) communities in German forests. *Insects* **2020**, *11*, 828.
164. Dekeirsschieter, J.; Frederick, C.; Verheggen, F.J.; Drugmand, D.; Haubruge, E. Diversity of forensic rove beetles (Coleoptera, Staphylinidae) associated with decaying pig carcass in a forest biotope. *J. Forensic Sci.* **2013**, *58*, 1032-1040.
165. Tumer, A.R.; Karacaoglu, E.; Namli, A.; Keten, A.; Farasat, S.; Akcan, R.; Sert, O.; Odabasi, A.B. Effects of different types of soil on decomposition: An experimental study. *Legal Med-Tokyo* **2013**, *15*, 149-156.
166. Payne J.A., King E.W.; Beinhart G. Arthropod succession and decomposition of buried pigs. *Nature*. **1968**, *219*, 1180.
167. Grisales, D.; Ruiz, M.; Villegas, S. Insects associated with exposed decomposing bodies in the Colombian Andean Coffee Region. *Rev. Bras. Entomol.* **2010**, *54*, 637-644.

168. Jarmusz, M.; Grzywacz, A.; Bajerlein, D. A comparative study of the entomofauna (Coleoptera, Diptera) associated with hanging and ground pig carcasses in a forest habitat of Poland. *Forensic Sci. Int.* **2020**, *309*, 110212.
169. Gomes, L.; Gomes, G.; Desuo, I. A preliminary study of insect fauna on pig carcasses located in sugarcane in winter in southeastern Brazil. *Med. Vet. Entomol.* **2009**, *23*, 155-159.
170. Madra, A.; Konwerski, S.; Matuszewski, S. Necrophilous Staphylininae (Coleoptera: Staphylinidae) as indicators of season of death and corpse relocation. *Forensic Sci. Int.* **2014**, *242*, 32-37.
171. Dawson, B.M.; Barton, P.S.; Wallman, J.F. Contrasting insect activity and decomposition of pigs and humans in an Australian environment: A preliminary study. *Forensic Sci. Int.* **2020**, *316*, 110515.
172. Eberhardt, T.L.; Elliot, D.A. A preliminary investigation of insect colonisation and succession on remains in New Zealand. *Forensic Sci. Int.* **2008**, *176*, 217-223.
173. Arnaldos, M.I.; Garcia, M.D.; Romera, E.; Presa, J.J.; Luna, A. Estimation of postmortem interval in real cases based on experimentally obtained entomological evidence. *Forensic Sci. Int.* **2005**, *149*, 57-65.
174. Barros De Souza, A.S.; Kirst, F.D.; Krueger, R.F. Insects of forensic importance from Rio Grande do Sul state in southern Brazil. *Rev. Bras. Entomol.* **2008**, *52*, 641-646.
175. Azwandi, A.; Keterina, H.N.; Owen, L.C.; Nurizzati, M.D.; Omar, B. Adult carrion arthropod community in a tropical rainforest of Malaysia: Analysis on three common forensic entomology animal models. *Trop. Biomed.* **2013**, *30*, 481-494.
176. Weithmann, S.; von Hoermann, C.; Degasperi, G.; Brandt, K.; Steiger, S.; Ayasse, M. Temporal variability of the rove beetle (Coleoptera: Staphylinidae) community on small vertebrate carrion and its potential use for forensic entomology. *Forensic Sci. Int.* **2021**, *323*, 110792.
177. Centeno N., Maldonado M.; Oliva A. Seasonal patterns of arthropods occurring on sheltered and unsheltered pig carcasses in Buenos Aires Province (Argentina). *Forensic Sci.Int.* **2002**, *126*, 63-70.
178. Paula Armani, A.; Dahinten, S.; Centeno, N. Fauna carrion associated to domestic pig (*Sus scrofa*) in a coastal environment in Chubut, Argentina. *Rev. Colomb. Entomol.* **2017**, *43*, 262-267.
179. Flores G. E.; Aballay F. H. Two *Evaniosomini* species (Coleoptera: Tenebrionidae) associated with decaying carcasses in Argentina, with remarks on the tribal assignment of *Achanius* Erichson. *Coleopt. Bull.* **2015**, *69*, 167-179.
180. Aballay F.H., Flores G.E., Silvestro V.A., Zanetti N.I.; Centeno N.D. An illustrated key to, and diagnoses of the species of Tenebrionidae (Coleoptera) associated with decaying carcasses in Argentina. *Ann. Zool.* **2016**, *66*, 703-726.
181. Hewadikaram K.A.; Goff M. L. Effect of carcass size on rate of decomposition and arthropod succession patterns. *Am. J. Foren. Med. Path.* **1991**, *12*, 235-240.
182. Al-Qahtni A., Mashaly A., Haddadi R.; Al-Khalifa M. Seasonal impact of heroin on rabbit carcass decomposition and insect succession. *Am. J. Foren. Med. Path.* **1991**, *12*, 235-240.
183. Shaalan, E.A.; El-Moaty, Z.A.; Abdelsalam, S.; Anderson, G.S. A preliminary study of insect succession in Al-Ahsaa Oasis, in the eastern region of the Kingdom of Saudi Arabia. *J. Forensic Sci.* **2017**, *62*, 239-243.
184. Mashaly A., Al-Khalifa M., Al-Qahtni A.; Alshehri A. Analysis of insects colonised on human corpses during autopsy in Riyadh, Saudi Arabia. *Entomol. Res.* **2020**, *50*, 351-360.
185. Levinson H.; Levinson A. Prionotheca coronata Olivier (Pimeliinae, Tenebrionidae) recognized as a new species of venerated beetles in the funerary cult of pre-dynastic and archaic Egypt. *J. Appl. Entomol.* **1996**, *120*, 577-585.
186. Al-Mekhlafi F.A. Beetles succession on different microhabitats of small mammals in Riyadh, Kingdom of Saudi Arabia. *Entomol. Res.* **2020**, *50*, 433-439.

187. Singh, S.; Yong, S.; Rahimi, R.; Singh, M.; Low, V.L.; Pittino, R.; Heo, C.C. First observation of *Afromorgus chinensis* (boheman, 1858) (Coleoptera: Trogidae) on a rabbit (*Oryctolagus cuniculus* L., 1758) carcass and its implications in forensic entomology. *Trop. Biomed.* **2023**, *40*, 370-374.
188. Struempher, W.P.; Farrell, J.; Scholtz, C.H. Trogidae (Coleoptera: Scarabaeoidea) in forensic entomology: occurrence of known and new species in Queensland, Australia. *Austral Entomol* **2014**, *53*, 368-372.
189. Diéguez, V.M.; Gómez, R.S. Aporte al conocimiento de las Trogidae (Coleoptera) de la Argentina. *Revista de la Sociedad Entomológica Argentina* **2004**, *63*, 92-95.
190. Ramos-Pastrana Y., Virguez-Diaz Y.; Wolff M. Insects of forensic importance associated to cadaveric decomposition in a rural area of the Andean Amazon, Caqueta, Colombia. *Acta Amazon.* **2018**, *48*, 126-136.
