# Supplementary material for: Research Status of Sarcosaprophagous Beetles as Forensic Indicators
Source: Insects. 2024 Sep 17;15(9):711. doi: 10.3390/insects15090711 (PMC11432003; doi:10.3390/insects15090711)
Supplement: Supplementary file 1 [file insects-15-00711-s001.zip › Table S3.pdf]

Table S3. Coleoptera that arrived at cadavers by chance. A total of 54 families and 111 species. **Ref:** References where the species was first recorded. **N:** The number of publications documenting the species in this data set.

**Family**	**Species**	**Ref**	**N**	**Types of cadaver or bait**
Agyrtidae	*Agyrtes castaneus*	[1]	1	deer
	Necrophilus hydrophiloides	[2]	1	pig
	Necrophilus subterraneus	[1]	1	deer
	Necrophilus sp.	[3]	1	pig
	unidentified	[4]	1	pig
Anobiidae	*Stegobium paniceum*	[5]	1	rat
	Stegobium sp.	[6]	1	pig
	unidentified	[7]	3	pig
Anthicidae	*Anthelephila imperatrix*	[8]	1	pig
	Hirticollis hispidus	[9]	1	pig
	Hirticollis quadriguttatus	[9]	1	pig
	Notoxus bifasciatus	[9]	1	pig
	Notoxus monoceros	[10]	1	fish
	Omonadus floralis	[11]	1	pig
	Stricticollis valgipes	[8]	1	pig
	unidentified	[12]	14	pig rat rabbit chicken
Archeocrypticidae	*Archeocrypticus topali*	[13]	1	pig
Bostrichidae	*Dinoderus minutus*	[14]	1	pig
	unidentified	[7]	3	pig rat
Buprestidae	unidentified	[15]	1	pig
Byrrhidae	*Byrrhus glabratus*	[1]	1	deer
	Byrrhus pilula	[1]	1	deer
	Simplocaria semistriata	[5]	1	rat
Cantharidae	*Cantharis* sp.	[3]	3	pig
	Metacantharis clypeata	[16]	1	rabbit
	Trypherus sp.	[17]	1	pig
	unidentified	[18]	6	pig
Cerambycidae	*Aerenea posticalis*	[17]	1	pig
	Compsocerus violaceus	[19]	1	pig
	Hesychotypa subfasciata	[17]	1	pig
	Rhagonycha sp.	[20]	1	rabbit
	unidentified	[21]	1	pig
Chrysomelidae	Alticinae sp.	[14]	2	pig
	Alticini sp.	[19]	1	pig
	Cassidini sp.	[19]	1	pig
	Chaetocnema sp.	[19]	1	pig
	Chalepus sanguinicollis	[19]	1	pig
	Chaotocnema sp.	[17]	1	pig
	Chrysolina bankii	[20]	1	rabbit
	Chrysolina fastuosa	[22]	1	pig

	Colaspis sp.	[19]	1	pig
	Cornulactica sp.	[19]	1	pig
	Desmogramma bivia	[19]	1	pig
	Diabrotica sp.	[19]	1	pig
	Diabrotica speciosa	[19]	1	pig
	Galerucinae sp.	[14]	2	pig
	Glyphocassis trilineata	[14]	1	pig
	Iucetima minor	[19]	1	pig
	Kuschelina vigintinitata	[19]	1	pig
	Longitarsus sp.	[17]	1	pig
	Megalognath sp.	[23]	1	pig
	Notosacantha sauteri	[14]	1	pig
	Omophoita octoguttata	[19]	1	pig
	Plagiosterna aenea	[24]	1	pig
	Psylliodes sp.	[25]	2	pig
	Stolas sp.	[17]	1	pig
	Systerna sp.	[17]	1	pig
	Timarcha goettingensis	[26]	1	pig
	Timarcha sp.	[20]	1	rabbit
	unidentified	[27]	8	human pig chicken rabbit dog
Clambidae	unidentified	[27]	1	chicken
Coccinellidae	*Brachiacantha* sp.	[17]	1	pig
	Coccinella novemnotata	[28]	1	rat
	Coccinella sp.	[29]	1	rat
	Cycloneda sanguinea	[19]	1	pig
	Epilachna cacica	[19]	1	pig
	Eriopis connexa	[19]	1	pig
	Hyperaspis erythrocephala	[22]	1	pig
	Hyperaspis festiva	[17]	1	pig
	Micraspis discolor	[30]	1	pig
	Nephus reunion	[31]	1	rabbit
	Rhyzobius litura	[26]	1	pig
	Scymus sp.	[23]	2	pig
	unidentified	[25]	6	pig dog
Corylophidae	*Orthoperus aequalis*	[25]	2	pig
	Orthoperus anxius	[25]	2	pig
	Sericoderus lateralis	[27]	3	pig chicken
	unidentified	[32]	2	pig
Cryptophagidae	*Antherophagus pallens*	[5]	2	pig rat
	Atomaria analis	[5]	1	rat
	Atomaria atricapilla	[5]	1	rat
	Atomaria basalis	[10]	1	fish
	Atomaria fuscata	[5]	1	rat
	Atomaria procerula	[5]	1	rat

	Atomaria scutellaris	[5]	1	rat
	Cryptophagus dentatus	[5]	1	rat
	Cryptophagus nitidulus	[5]	1	rat
	Cryptophagus pallidus	[5]	1	rat
	Cryptophagus pilosus	[5]	2	fish rat
	Cryptophagus pseudodentatus	[10]	1	fish
	Cryptophagus setulosus	[5]	2	fish rat
	Cryptophagus sporadum	[5]	1	rat
	unidentified	[27]	5	pig chicken rat dog
Cucujidae	unidentified	[33]	1	pig
Curculionidae	*Coccotrypes* sp.	[34]	1	pig
	Episomus turritus	[35]	1	pig
	Hylobius abietis	[1]	1	deer
	Ips typographus	[1]	1	deer
	Naupactini sp.	[19]	1	pig
	Nicentrus decipiens	[22]	1	pig
	Otiorhynchus laevigatus	[26]	1	pig
	Otiorhyncus sp.	[36]	1	pig
	Phloeborus punctatorugosus	[22]	2	pig
	Phyllobius arborator	[1]	1	deer
	Plinthus tischeri	[1]	1	deer
	Romualdius scaber	[37]	3	deer
	Scolytinae sp.	[38]	1	cat
	Stephanoderes sp.	[39]	1	cat
	Stenopelmus sp.	[40]	1	pig
	Xyleborus sp.	[41]	3	pig human
	unidentified	[27]	11	pig chicken dog
Dryophthoridae	*Sipalinus gigas*	[8]	1	pig
	Sitophilus zeamais	[14]	1	pig
Dryopidae	*Helichus* sp.	[40]	1	pig
	unidentified	[17]	1	pig
Elateridae	*Agrypnus murinus*	[26]	1	pig
	Athous subfuscus	[5]	1	rat
	Chiagosnius obscuripes	[42]	1	pig
	Conoderus bellus	[32]	1	alligator
	Conoderus sp.	[43]	1	pig
	Ctenicera comes	[3]	1	pig
	Drasterius bimaculatus	[44]	1	pig
	Pyrophorini sp.	[3]	1	pig
	unidentified	[28]	11	pig rat cat chicken
Elmidae	*Heterelmis* sp.	[6]	1	pig
	Heterlimnius sp.	[40]	1	pig
	Optioservus sp.	[40]	1	pig
	Stenelmis sp.	[40]	1	pig

	unidentified	[22]	2	pig
Endomychidae	*Ancylopus pictus asiaticus*	[8]	1	pig
	Lycoperdina bovistae	[5]	1	rat
	Mycetina cruciata	[1]	1	deer
Eucinetidae	unidentified	[33]	1	pig
Gyrinidae	*Gyrinus* sp.	[6]	1	pig
Hallplidae	*Pehodytes* sp.	[45]	1	pig
Hybosoridae	*Chaetodus exaratus*	[46]	1	rabbit
	unidentified	[19]	1	pig
Laemophloeidae	unidentified	[47]	1	pig
Lampyridae	*Lampyris noctiluca*	[1]	1	deer
	Lucidota sp.	[19]	1	pig
	Lycluuris rufa	[24]	1	pig
	Pollaclasis sp.	[48]	1	pig
	Photinus vittatus	[41]	1	pig
	Photuris sp.	[48]	1	pig
	unidentified	[14]	3	pig
Languriidae	*Cryptophilus integer*	[49]	1	pig
	Cryptophilus sp.	[25]	1	pig
Lathridiidae	*Aridius nodifer*	[5]	1	rat
	Corticaria impressa	[5]	1	rat
	Corticaria longicornis	[5]	1	rat
	Cortinicara gibbosa	[5]	1	rat
	Dienerella vincenti	[5]	1	rat
	Enicmus transversus	[5]	1	rat
	Lathridius lardarius	[50]	1	rabbit
	Melanophthalma curticollis	[5]	1	rat
	Migneauxia orientalis	[51]	1	pig
	Stephostethus angusticollis	[5]	1	rat
	Stephostethus lardarius	[5]	1	rat
	Stephostethus rugicollis	[5]	1	rat
	unidentified	[33]	4	pig human
Leiodidae	*Hydnobius* sp.	[17]	1	pig
Limnychidae	unidentified	[47]	1	pig
Lucanidae	unidentified	[33]	1	pig
Lycidae	*Dictyoptera aurora*	[5]	1	rat
	unidentified	[48]	1	pig
Lyctidae	unidentified	[41]	1	pig
Lymexylidae	unidentified	[38]	1	pig
Malachiidae	unidentified	[25]	1	pig
Melandryidae	unidentified	[25]	2	pig
Meloidae	*Meloe lunata*	[52]	2	pig sheep
	unidentified	[33]	1	pig

Melolonthidae	unidentified	[48]	4	pig
Melyridae	*Astylus* sp.	[13]	1	pig
	Dasytes virens	[31]	2	rabbit
	Dasyrhadus sp.	[48]	1	pig
	unidentified	[25]	3	pig
Monotomidae	*Monotoma picipes*	[39]	2	cat bear
	Rhizophagus bipustulatus	[5]	1	rat
	Rhizophagus dispar	[5]	1	rat
	Rhizophagus parallelocollis	[53]	1	human
	Rhizophagus sp.	[54]	1	pig
	unidentified	[33]	2	pig
Mordellidae	*Mordellidae* sp.	[8]	1	pig
Mycetophagidae	*Mycetophagidae* sp.	[55]	1	impala
	Typhaea stercorea	[39]	2	pig cat
	unidentified	[33]	4	pig
Noteridae	*Canthydrus laetabilis*	[56]	2	pig rabbit
Oedemeridae	unidentified	[48]	1	pig
Passalidae	*Passalus interruptus*	[22]	1	pig
Phalacridae	unidentified	[33]	2	pig
Phengodidae	unidentified	[21]	2	pig
Ptilodactylidae	unidentified	[14]	2	pig
Ptinidae	unidentified	[25]	1	pig
Scraptiidae	*Anaspis rufilabris*	[1]	1	deer
	unidentified	[25]	3	pig
Scydmaenidae	*Cephennium majus*	[5]	1	rat
	Euconnus pubicollis	[5]	1	rat
	Neuraphes elongatulus	[5]	1	rat
	unidentified	[25]	4	pig rat
Silvanidae	unidentified	[27]	4	pig chicken dog
Sphaeritidae	*Sphaerites glabratus*	[1]	1	pig

References

1. von Hoermann, C.; Lackner, T.; Sommer, D.; Heurich, M.; Benbow, M.E.; Mueller, J. Carcasses at Fixed Locations Host a Higher Diversity of Necrophilous Beetles. *Insects* **2021**, *12*.
2. Dillon, L.C. Insect succession on carrion in three biogeoclimatic zones of British Columbia. Master’s Thesis, Simon Fraser University, British Columbia, Canada, 1997.
3. VanLaerhoven, S.L. Successional biodiversity in insect species on buried carrion in the Vancouver and Cariboo regions of British Columbia. Master’s Thesis, Simon Fraser University, British Columbia, Canada, 1997.
4. Eberhardt, T.L.; Elliot, D.A. A preliminary investigation of insect colonisation and succession on remains in New Zealand. *Forensic Sci. Int.* **2008**, *176*, 217-223.
5. Kocarek, P. Decomposition and coleoptera succession on exposed carrion of small mammal in Opava, the Czech Republic. *Eur. J. Soil Biol.* **2003**, *39*, 31-45.

6. Barrios, M.; Wolff, M. Initial study of arthropods succession and pig carrion decomposition in two freshwater ecosystems in the Colombian Andes. *Forensic Sci. Int.* **2011**, *212*, 164-172
7. Davis, J.B. Decomposition Patterns in Terrestrial and Intertidal Habitats on O'ahu Island and Coconut Island (Moku O Loe), Hawai'i. Master's Thesis, University of Hawai'i at Manoa, Honolulu, HI, USA, 1998.
8. Park, S.; Lee, J.; Woo, D.; Ji, B.; Moon, T. Insect diversity and succession patterns on pig cadavers in Changwon, South Korea. *Entomol. Res.* **2022**, *52*, 241-250.
9. Diaz-Aranda L.M., Martin-Vega D., Gomez-Gomez A., Cifrian B.; Baz A. Annual variation in decomposition and insect succession at a periurban area of central Iberian Peninsula. *J. Forensic Leg Med*. **2018**, *56*, 21-31.
10. Ulrich, W.; Zalewski, M.; Komosinski, K. Diversity of carrion visiting beetles at rural and urban sites. *Community Ecol.* **2007**, *8*, 171-181.
11. Hu, G.; Kang, C.; Zhu, R.; Guo, Y.; Li, L.; Wang, Y.; Zhang, Y.; Wang, Y.; Wang, J. A preliminary study of body decomposition and arthropod succession in an arid area in northwest China during summer. *J. Med. Entomol.* **2023**, *60*, 306-315.
12. Tantawi T. I., Elkady E. M., Greenberg B.; ElGhaffar H. A. Arthropod succession on exposed rabbit carrion in Alexandria, Egypt. *J. Med. Entomol.* **1996**, *33*, 566-580.
13. Zanetti, N.I.; Visciarelli, E.C.; Centeno, N.D. Trophic roles of scavenger beetles in relation to decomposition stages and seasons. *Rev. Bras. Entomol.* **2015**, *59*, 132-137.
14. Lyu Z., Wan L., Yang Y., Tang R.; Xu L. A checklist of beetles (Insecta, Coleoptera) on pig carcasses in the suburban area of southwestern China: A preliminary study and its forensic relevance. *J. Forensic Leg. Med.* **2016**, *41*, 42-48.
15. Bonacci, T.; Mendicino, F.; Bonelli, D.; Carlomagno, F.; Curia, G.; Scapoli, C.; Pezzi, M. Investigations on arthropods associated with decay stages of buried animals in Italy. *Insects* **2021**, *12*.
16. Chapman R.F.; Sankey J. The larger invertebrate fauna of 3 rabbit carcasses. *J. Anim. Ecol.* **1955**, *24*, 395-402.
17. Mise K. M., de Almelda L. M.; Moura M. O. A study of the Coleoptera (Insecta) fauna that inhabits *Sus scrofa* L carcass in Curitiba, Parana. *Rev. Bras. Entomol.* **2007**, *51*, 358-368.
18. Watson E.J.; Carlton C.E. Insect succession and decomposition of wildlife carcasses during fall and winter in Louisiana. *J. Med. Entomol.* **2005**, *42*, 193-203.
19. Ries, A.C.R.; Costa-Silva, V.; Dos Santos, C.F.; Blochtein, B.; Thyssen, P.J. Factors affecting the composition and succession of beetles in exposed pig carcasses in southern Brazil. *J. Med. Entomol.* **2021**, *58*, 104-113.
20. Taleb, M.; Tail, G.; Djedouani, B.; Acikgoz, H.N. Impact of plastic wrapping on carcass decomposition and arthropod colonisation in northern Africa during spring. *Sci. Justice* **2022**, *62*, 117-127.
21. Carvalho, L.; Thyssen, P.J.; Linhares, A.X.; Palhares, F. A checklist of arthropods associated with pig carrion and human corpses in southeastern Brazil. *Mem. I. Oswaldo Cruz* **2000**, *95*, 135-138.
22. Ramos-Pastrana Y., Virguez-Diaz Y.; Wolff M. Insects of forensic importance associated to cadaveric decomposition in a rural area of the Andean Amazon, Caqueta, Colombia. *Acta Amazon.* **2018**, *48*, 126-136.
23. Ekanem M. S.; Dike M. C. Arthropod succession on pig carcasses in southeastern Nigeria. *Papéis Avulsos de Zoologia (São Paulo)*. **2010**, *50*, 561-570.
24. Zheng, Z.; Yin, M. A Study on the arthropod succession in exposed pig carrion. *Journal of Life Science* **2008**, *18*, 1400-1409.
25. Castro, C.B.D.P. Seasonal carrion Diptera and Coleoptera communities from Lisbon (Portugal) and the utility of forensic entomology in legal medicine. Master's Thesis, Universidade de Lisboa , Portugal, 2011.
26. Anton, E.; Niederegger, S.; Beutel, R.G. Beetles and flies collected on pig carrion in an experimental setting in Thuringia and their forensic implications. *Med. Vet. Entomol.* **2011**, *25*, 353-364.
27. Arnaldos, M.I.; Romera, E.; Presa, J.J.; Luna, A.; Garcia, M.D. Studies on seasonal arthropod succession on

carrion in the southeastern Iberian Peninsula. *Int. J. Legal Med.* **2004**, *118*, 197-205.
28. De Jong, G.D.; Hoback, W.W. Effect of investigator disturbance in experimental forensic entomology: succession and community composition. *Med. Vet. Entomol.* **2006**, *20*, 248-258.
29. Al-Mekhlafi F.A. Beetles succession on different microhabitats of small mammals in Riyadh, Kingdom of Saudi Arabia. *Entomol. Res.* **2020**, *50*, 433-439.
30. Moreau G., Ramal A.F., Letana S.D.; Horgan F.G. Death in the paddy field: Carcass decomposition and associated arthropods in subunits of a rice field landscape. *Forensic Sci.Int.* **2022**, *335*, 111288.
31. Al-Khalifa M., Mashaly A.; Al-Qahtni A. Impacts of antemortem ingestion of alcoholic beverages on insect successional patterns. *Saudi J. Biol. Sci.* **2021**, *28*, 685-692.
32. Watson E. J. and Carlton C. E. Spring succession of necrophilous insects on wildlife carcasses in Louisiana. *J. Med. Entomol.* **2003**, *40*, 338-347.
33. Gill, G.J. Decomposition and arthropod succession on above ground pig carrion in rural Manitoba. Master's Thesis, University of Manitoba, Manitoba, Canada, 2005.
34. Mashaly, A.M.A. Entomofaunal succession patterns on burnt and unburnt rabbit carrion. *J. Med. Entomol.* **2016**, *53*, 296-303.
35. Zheng, Z.; Yin, M. Arthropod succession and decomposition patterns of pig carrions varying with the exposed extent of the carrions. *Journal of Life Science* **2011**, *21*, 1168-1175.
36. Ivorra T., Rahimi R., Goh T. G., Azmiera N., Nur-Aliah N. A.; Low V. L. et al. First record of *Diamesus osculans* (Vigors, 1825) (Coleoptera: Silphidae) colonization on a human corpse. *Int. J. Legal Med.*, **2023**, *138*, 677–683.
37. Melis, C.; Teurlings, I.; Linnell, J.; Andersen, R.; Bordoni, A. Influence of a deer carcass on Coleopteran diversity in a Scandinavian boreal forest: a preliminary study. *Eur. J. Wildlife Res.* **2004**, *50*, 146-149.
38. Mayer, A.C.G.; Vasconcelos, S.D. Necrophagous beetles associated with carcasses in a semi-arid environment in Northeastern Brazil: Implications for forensic entomology. *Forensic Sci. Int.* **2013**, *226*, 41-45.
39. Early, M. Arthropod succession patterns in exposed carrion in Hawaii. Master's Thesis, University of Hawai'i at Manoa, HI, USA, 1984.
40. Hobischak, N.R. Freshwater invertebrate succession and decompositional studies on carrion in British Columbia. Master's Thesis, Simon Fraser University, British Columbia, Canada, 1997.
41. Guarin Vargas, E.G. Insectos de importancia forense asociados a la descomposición cadavérica del cerdo Sus domesticus, expuesto a sol, sombra total y sombra parcial, en Mayagüez, Puerto Rico. Master's Thesis, University of Puerto Rico, Mayaguez, USA, 2006.
42. Wang, J.; Li, Z.; Chen, Y.; Chen, Q.; Yin, X. The succession and development of insects on pig carcasses and their significances in estimating PMI in south China. *Forensic Sci. Int.* **2008**, *179*, 11-18.
43. Perez, S.P.; Duque, P.; Wolff, M. Successional behavior and occurrence matrix of carrion-associated arthropods in the urban area of Medellin, Colombia. *J. Forensic Sci.* **2005**, *50*, 448-454.
44. Tumer, A.R.; Karacaoglu, E.; Namli, A.; Keten, A.; Farasat, S.; Akcan, R.; Sert, O.; Odabasi, A.B. Effects of different types of soil on decomposition: An experimental study. *Legal Med-Tokyo* **2013**, *15*, 149-156.
45. Vance G. M., Vandyk J. K.; Rowley W. A. Device for sampling aquatic insects associated with carrion in water. *J. Forensic Sci.* **1995**, *40*, 479-482.
46. Correa, R.C.; Almeida, L.M.; Moura, M.O. Coleoptera associated with buried carrion: potential forensic importance and seasonal composition. *J. Med. Entomol.* **2014**, *51*, 1057-1066.
47. Ramos-Pastrana Y., Rafael J. A.; Wolff M. Pig (*sus scrofa*) decomposition in lotic and lentic aquatic systems as tool for determination a postmortem submersion interval in the Andean Amazon, Caquetá, Colombia. *Boletín Científico. Centro de Museos. Museo de Historia Natural* **2019**, *23*, 55-72.

48. Martinez, E.; Duque, P.; Wolff, M. Succession pattern of carrion-feeding insects in Paramo, Colombia. *Forensic Sci. Int.* **2007**, *166*, 182-189.
49. Prado E Castro C., Dolores Garcia M., Da Silva P. M., Faria E Silva I.; Serrano A. Coleoptera of forensic interest: A study of seasonal community composition and succession in Lisbon, Portugal. *Forensic Sci.Int.* **2013**, *232*, 73-83.
50. Bourel B., Martin-Bouyer L., Hedouin V., Cailliez J. C., Derout D.; Gosset D. Necrophilous insect succession on rabbit carrion in sand dune habitats in northern France. *J. Med. Entomol.* **1999**, *36*, 420-425.
51. Zou T., Feng D., Huang G., Sun D.; Dai S. Species composition and succession of necrophagous insects on small buried baits in China. *J. Med. Entomol.* **2022**, *59*, 1182-1190.
52. Tembe D., Malatji M. P.; Mukaratirwa S. An exploratory study of beetles and flies of forensic importance on sheep carrion in KwaZulu-Natal province of South Africa. *Afr. Entomol.* **2021**, *29*, 590-601.
53. Dekeirsschieter J., Frederickx C., Verheggen F. J., Boxho P.; Haubruge E. Forensic entomology investigations from doctor Marcel Leclercq (1924-2008): a review of cases from 1969 to 2005. *J. Med. Entomol.* **2013**, *50*, 935-954.
54. Hobischak, N.R.; VanLaerhoven, S.L.; Anderson, G.S. Successional patterns of diversity in insect fauna on carrion in sun and shade in the Boreal Forest Region of Canada, near Edmonton, Alberta. *Can. Entomol.* **2006**, *138*, 376-383.
55. Ellison G. The effect of scavenger mutilation on insect succession at impala carcasses in Southern Africa. *J. Zool.* **1990**, *220*, 679-688.
56. Dalal, J.; Sharma, S.; Bhardwaj, T.; Dhattarwal, S.K.; Verma, K. A seasonal study of the decomposition pattern and insects on submerged rabbit carcases. *Orient. Insects* **2021**, *55*, 280-292.
